# Epigenetic Regulation of Plant Tolerance to Salt Stress by Histone Acetyltransferase GsMYST1 From Wild Soybean

**DOI:** 10.3389/fpls.2022.860056

**Published:** 2022-05-25

**Authors:** Peng Feng, Xiaohuan Sun, Xiaodong Liu, Yuqiu Li, Qi Sun, Haoran Lu, Minglong Li, Xiaodong Ding, Yingshan Dong

**Affiliations:** ^1^Key Laboratory of Agricultural Biological Functional Genes, College of Life Science, Northeast Agricultural University, Harbin, China; ^2^College of Life Science, Leshan Normal University, Leshan, China; ^3^Soybean Research Institute, Jilin Academy of Agricultural Sciences, Changchun, China; ^4^Mudanjiang Branch of Heilongjiang Academy of Agricultural Sciences, Mudanjiang, China; ^5^Key Laboratory of Soybean Biology of Chinese Education Ministry, Northeast Agricultural University, Harbin, China

**Keywords:** soybean, abiotic stress, SnRK1 kinase, histone acetylation, NAC transcription factor

## Abstract

Salt stress is one of the most devastating environmental factors threatening soybean growth and yield. However, the molecular link between salt stress and epigenetics has not been well-elucidated in soybean. In this study, from the wild soybean cDNA library, we isolated a GsSnRK1 kinase interacting protein (GsMSTY1) which is phylogenetically homologous with histone acetyltransferase MYST family with unknown function. *GsMSTY1* gene is dominantly expressed in wild soybean roots and is highly responsive to abiotic stresses. GsMYST1 was able to be phosphorylated at the Ser44 site by GsSnRK1 and demonstrated *in vivo* acetyltransferase activity in transgenic soybean roots revealed by an anti-H4ace antibody. A transcription factor protein GsNAC83 was identified to interact with both GsMYST1 and GsSnRK1, and GsNAC83 could recruit the GsMYST1-GsSnRK1 module to *COR15B* gene promoter determined by ChIP-qPCR assay. To dissect the molecular functions of this ternary complex, we treated the transgenic soybean roots with salt stress and found that the stress could activate GsSnRK1, and the activated GsSnRK1 subsequently phosphorylated GsMYST1 to enhance its acetyltransferase activity which may epigenetically promote the target gene expression. To explore the physiological functions, we coexpressed *GsSnRK1* and *GsMYST1* genes in soybean hairy roots and found that only *GsSnRK1*(wt)/*GsMYST1*(wt) but not the mutant genes could promote soybean resistance to salt stress, implicating that phosphorylation of GsMYST1 is required for it to acetylate histone H4 on the target genes to upregulate expression of the stress-related genes. Our data shed new light on the functions of the GsSnRK1-GsMYST1-GsNAC83 module and its regulatory mechanism on plant tolerance to abiotic stresses.

## Highlights

- GsSnRK1 and GsMYST1 coordinately regulate soybean stress resistance.

## Introduction

Soybean (*Glycine max*) is an important economic crop, which provides protein and oil for human and animals and enhances soil nutrients through nitrogen fixation *via* plant-microorganism symbiosis. Owing to long-term artificial selection, the cultivated soybeans have lost many functional genes to adapt to varying environments, that is to say, soybeans are sensitive to many biological and abiotic stresses, such as drought, salt, and cold stresses, compared with the other crops. However, wild soybeans (*Glycine soja*) are close relatives of cultivated soybeans and possess special genes to adapt to harsh environmental conditions (Xie et al., 2019). Therefore, exploring the new genes from wild soybeans for the improvement of soybean adaptabilities becomes more and more important.

The response and adaptation of plants to environmental stresses are achieved through transcriptional reprogramming, which is regulated by a set of differentially activated or repressed transcription factors. The gene differential expression is usually caused by the changes in specific epigenetic modification levels on the regulatory regions of the target genes through stress signal transduction (Kouzarides, 2007; Nakashima et al., 2014). Eukaryotic genomic DNA is packaged around the octamers of histones to form the basic structural units of chromatin, namely, nucleosome. Chromatin is the functional template for a series of key biological processes, such as DNA replication, DNA damage repair, recombination, and transcription (Latrasse et al., 2008). Covalent modifications of the N-terminal tails of core histones affect the localization and compression of nucleosomes, so they play key roles in chromatin remodeling and gene regulation, including acetylation, methylation, phosphorylation, ubiquitination, sumoylation, and poly-ADP-ribosylation (Liu et al., 2014). Among these modifications, histone acetylation and deacetylation play important roles in the regulation of gene expression in plants, as the key switches for the transition between permissive and repressive states of chromatin domains (Berger, 2007).

The antagonistic effects of histone acetyltransferase (HAT) and histone deacetylase (HDAC) maintained the homeostasis of histone acetylation in the nucleosome. In *Arabidopsis thaliana*, the HAT group contains 12 members, which are divided into 4 groups on the basis of sequence similarity and characteristics: GNAT (Gcn5-related N-acetyltransferase), p300/CBP, TAFII250, and MYST (MOZ, YbF2, Sas2, and Tip60-like) families (Pandey et al., 2002). In recent years, the research in plants mainly focused on the GNAT family histone acetyltransferases. Mounting evidence have linked specific histone acetyltransferases to transcriptional regulation in plants. For instance, GCN5 histone acetyltransferase plays a role in the regulation of numerous processes, including cold tolerance, floral development, embryonic cell-fate patterning, and light responsiveness (Kumar et al., 2021). Although the GNAT family has been extensively studied in plant stress resistance, the functions of MYST family histone acetyltransferases are still elusive.

The MYST family of HAT is highly conserved in eukaryotes. The members play critical roles in gene-specific transcription regulation, DNA repair, and replication. Participation in such basic nuclear functions suggests that alteration of these MYST HATs might lead to multiple-growth defects (Pillus, 2008). In recent years, the studies on plant MYST family histone acetyltransferases mainly focused on growth and development but few on their roles in abiotic stresses. In *Arabidopsis*, there are two functionally redundant MYST family histone acetyltransferase members, namely, HAM1 and HAM2. Genetic analysis and cytological study revealed that *ham1/ham2* double mutation-induced severe defects in the formation of male and female gametophytes, resulting in an arrest of the mitotic cell cycle at the early stages of gametogenesis (Latrasse et al., 2008); HAM1 and HAM2 play critical roles in regulating flowering time through epigenetic modification of the H4K5ace status within chromatins of *FLC* and *MADS-box Affecting Flowering* genes 3/4 (*MAF3/4*) and silencing of *HAM1* and *HAM2* genes causing early flowering and reduced fertility (Xiao et al., 2013). In *Arabidopsis*, both HAM1 and HAM2 have H4K5 specific HAT activity *in vitro* and *in vivo* (Earley et al., 2007; Xiao et al., 2013).

Sucrose non-fermentation-related protein kinases (SnRKs) are a class of Ser/Thr protein kinases widely existing in plants. It has high homology with *Saccharomyces cerevisiae* carbon source regulated protein kinase (SNF1) and animal AMPK (AMP-activating protein kinase) protein kinase in sequence and function (Halford and Hardie, 1998). According to the protein structures and functions, SnRKs can be divided into three subfamilies, namely, SnRK1, SnRK2, and SnRK3. Members of each family perform different functions in regulating plant growth and physiological metabolism. SnRK1s are much less studied than SnRK2s and SnRK3s, but the accumulated studies show that SnRK1s are the main energy sensors in the metabolic signal network that controls plant growth and development and tolerance to biotic/abiotic stresses (Crozet et al., 2014). In yeast, Snf1 protein kinase can be activated by salt alkali stress and oxidative stress. *Snf1*Δ mutant showed sensitivity to salt and high pH, indicating that the Snf1 kinase was directly involved in the regulation of salt alkali stress in yeast (Hong and Carlson, 2007). The *Snf1*Δ mutant complemented with *Secale cereale SnRK1* kinase gene can make yeast utilize non-fermentable carbon sources, such as ethanol and glycerin, which indicates that plant SnRK1 kinase can replace yeast Snf1 kinase in sugar signal transduction pathway (Alderson et al., 1991). This experiment proves that the function of plant homologs is conservative, and SnRK1 participates in energy metabolism regulation. *SnRK1* genes from tobacco (*Nicotiana tabacum*), potato (*Solanum tuberosum*), *A. thaliana*, and wild soybean (unpublished data) can also complement *Snf1*Δ mutants (Crozet et al., 2014). We also found that wild soybean GsSnRK1 can interplay with transcription factor GsERF7 from wild soybean to regulate soybean stress resistance (Feng et al., 2020). Phosphorylation by protein kinases and acetylation by histone acetyltransferases may be coupled to participate in gene regulation. For example, SnRK1-mediated C/S1-basic leucine zipper transcription factor (C/S1-bZIP) signaling activates alternative mitochondrial metabolic pathways to ensure plant survival in extended darkness. S1-bZIPs (bZIP1, bZIP2, bZIP11, bZIP44, and bZIP53) act as SnRK1-dependent regulators that directly regulate transcription *via* binding to G-box promoter elements. S1-bZIPs do not physically interact with SnRK1, but C-bZIPs (bZIP9, bZIP10, bZIP25, and bZIP63) as the *in vivo* transcription factor can bridge SnRK1 to S1-bZIPs and then form a ternary complex. Thus, the C/S1-bZIP-SnRK1 complex interacts with the histone acetylation machinery to remodel chromatin and facilitate transcription (Pedrotti et al., 2018).

NAC transcription factors are one of the largest transcription factor families in plants. *Arabidopsis* genome has ~110 NAC members, most of which are involved in different developmental processes and biotic and abiotic stress responses. In *Arabidopsis*, a NAC transcription factor named VNI2 transcription factor regulates cold regulation and dehydration genes by binding to the target gene promoters to mediate signal crosstalk between salt stress response and leaf senescence (Yang et al., 2011). In *Populus trichocarpa*, under drought stress conditions, the expression of the *AREB1* transcription factor is induced. AREB1 interacts with the ADA2b-GCN5 HAT complex and recruits the proteins to *PtrNAC006, PtrNAC007*, and *PtrNAC120* genes through binding to ABRE motifs, resulting in enhanced H3K9ac and RNA Pol II enrichment for activating the expression of the *PtrNAC006, PtrNAC007*, and *PtrNAC120* genes (Li et al., 2019).

In this study, we determined the physical and genetic interaction of GsSnRK1 and an MYST family histone acetyltransferase, GsMYST1 from wild soybean, and reported the mechanism of how GsSnRK1, GsMYST1, and GsNAC83 form a heterotrimeric complex to cooperatively regulate the expression *COR15B*, a downstream target gene of transcription factor GsNAC83. Finally, we proposed a model of how GsSnRK1-GsMSYT1-GsNAC83 synergistically modulates plant resistance to salt stress, which provides a broader view of the novel signaling pathway of SnRK1 protein kinase.

## Materials and Methods

### Plant Materials

The seeds of wild soybean (*G. soja* G07256) were collected from saline–alkaline fields in Baicheng County, Jilin Province, and were maintained in the lab. The seeds were treated with sulfuric acid and scarified before germination and then stratified on a wet filter paper at 4°C in the dark for 3 days to break the seed dormancy. Wild soybean seedlings were then cultured in 1/4 Hoagland nutrient solution in a plant growth chamber. The growth conditions were as follows: 24°C and relative humidity of 60%. The light cycle is 16 h of light and 8 h of darkness. The germinated seeds were irrigated and sown in nursery soil for 3 weeks. Conditions were the same as for growth in the Hoagland medium. The stress treatment was carried out by irrigating plants with 150 mM NaCl for a period of time. Plant materials were collected and stored at −80°C for further application.

### RNA Extraction, cDNA Synthesis, and Quantitative Real-Time PCR

Plant total RNA samples were extracted using TRIzol reagent by following the manufacturer's instructions, and cDNA was synthesized using the Transcript ALL-in-One First-Strand cDNA Synthesis SuperMix Kit (Transgene, China). The qRT-PCR analyses were performed using TransStart Top Green qPCR Supermax Kit (Transgene, China). The 2^−Δ*ΔCT*^ method was used to analyze the data of the samples. The used primers are listed in Supplementary Table S1.

### Protoplast Preparation and Transformation

The 3-week-old young *Arabidopsis* leaves were cut into 0.5–1 mm strips with fresh razor blades without wounding. The leaf strips were submerged in an enzyme solution (1.5% cellulose R10, 0.4% macerozyme R10, 0.4 M mannitol, 20 mM KCl, 20 mM MES, pH 5.5, 10 mM CaCl_2_, and 0.1% BSA) in a Petri dish and were put into to a vacuum desiccator for 5–30 min. The samples were further digested for 60–90 min with gentle shaking. The suspension was filtered through a 35- to 75-μm nylon mesh. The pass-through was spun at 100 × *g* to pellet the protoplasts in a round-bottomed tube for 1–2 min. The protoplast pellets were resuspended in MMg solution (9% mannitol, 15 mM MgCl_2_, and 4 mM MES) to a density of 1.0 × 10^7^ protoplasts/ml; 5 μg of plasmid was mixed gently with 200 μl of protoplasts. Freshly prepared PEG4000 solution (9% mannitol, 100 mM CaCl_2_) was immediately added and mixed by gentle inversion to obtain a 25% final PEG4000 concentration. The mixture was incubated at room temperature for 20 min. After incubation, the transfection mixture was gently diluted with three volumes of W5 solution (154 mM NaCl, 125 mM CaCl_2_, 5 mM KCl, and 2 mM MES, pH 5.8). The transfected protoplasts were centrifuged (80 × *g*, 2 min) twice and then were resuspended in 300–500 μl of WI solution (4 mM MES, 9% mannitol, and 20 mM KCl, pH 5.8) and incubated overnight at room temperature in dark to induce the target protein expression.

### Yeast One-Hybrid and Two-Hybrid Screens

The cDNA library for yeast two-hybrid (Y2H) was constructed using the method described previously (Song et al., 2019; Feng et al., 2020). The Y2H screening was carried out according to the method (Ding et al., 2007; Song et al., 2019). HF7c cells containing bait vector pGBKT7-GsMYST1 and Y187 cells containing wild soybean cDNA library were mixed for yeast mating. The obtained diploid yeast zygotes (>10^6^) were spread on SD/-Leu-Trp-His medium supplied with 5 mM 3-AT (3-amino-1,2,4-triazole). The prey plasmid was extracted from the yeast clone and sequenced. Yeast one-hybrid (Y1H) analysis was carried out according to the user manual of the Y1H library screening system (Clonetek, USA). The gene GsNAC83 was inserted into the vector pGADT7, the synthesized DNA sequence was inserted into the vector pHIS2.1, then cotransformed into Y187 yeast, and spread on SD/-Leu-Trp-His medium supplied with 10 mM 3-AT.

### Bimolecular Fluorescence Complementation and Co-Immunoprecipitation Assays

The full-length GsMYST1 and GsSnRK1 or GsMYST1 and GsNAC83 or GsNAC83 and GsSnRK1 genes were cloned into the upstream of YFP N173 and YFP C155 in pPBEL-bimolecular fluorescence complementation (BiFC) vector (Song et al., 2019; Feng et al., 2020). The recombinant plasmids were transfected into *Arabidopsis* protoplasts to express split YFP protein for BiFC analysis for 12–15 h. The YFP signal was observed under a confocal microscope at a 500-nm excitation wavelength. Protein co-immunoprecipitation (co-IP) assays were carried out according to the described protocol.

### Prokaryotic Expression and Purification of Recombinant Proteins

The recombinant proteins were expressed and purified according to the Novagen pET system manual. GsMYST1 or GsSnRK1 and its mutants were inserted into the pET32b vector and transformed into *Escherichia coli* BL21 competent cells to express the recombinant protein. The protein was purified with Ni-NTA beads, and the eluted protein was dialyzed in Tris-buffered saline (TBS) buffer to remove imidazole.

### Protein Phosphorylation Assay

The purified GsSnRK1 and its substrate were mixed in kinase assay buffer (20 mM HEPES, pH 7.6, 25 mM β-glycerophosphate, 0.1 mM Na_3_VO_4_, 4 mM NaF, 2 mM MnCl_2_, 10 mM MgCl_2_, and 10 μM non-radioactive ATP) and incubated at 30°C for 30 min. Dephosphorylation was carried out by treating the protein samples with calf intestine phosphatase (CIP). The protein samples were resolved in normal sodium dodecyl-sulfate polyacrylamide gel electrophoresis (SDS-PAGE) gel or SDS-PAGE gel with 12% acrylamide plus 25 μM phos-tag (AAL-107, Wako) and 25 μM MnCl_2_ to perform phos-tag assays according to the described method (Kinoshita et al., 2006). For phos-tag assays, the gels were washed with blotting buffer containing 20 mM ethylenediaminetetraacetic acid (EDTA) for 1 h to chelate Mn^2+^. Proteins were transferred to the polyvinylidene difluoride (PVDF) membrane for Western blots (WB) using certain antibodies. Phospho-(Ser/Thr) PKD substrate antibody (cat no: 4381S) was purchased from Cell Signaling Technology (MA, USA). pPKD substrate antibody can recognize the phosphorylated serine/threonine residue with R/K at −3 and L at −5, preferring proline at the −1 position within the motif (LxR/KxFpSxxxx). Phospho-AMPKα(Thr172) (cat. no: 2535S) was also purchased from Cell Signaling Technology. This antibody recognizes the phosphorylated Thr176 in GsSnRK1 as well as the phosphorylated Thr172 in mammalian AMPK and Thr210 in yeast SNF1.

### Induction and Transformation of Hairy Roots in Soybean Plants

The wild-type *GsSnRK1, GsMYST1*, and their mutant genes with different tags were cloned into plant binary vectors. The constructs were transformed into *Agrobacterium rhizogenes* K599 strain. The seeds of soybean Dongnong 50 were sowed and germinated in wet vermiculite 1–2 cm deep in nutritive pots. After emerging from the soil, the seedlings were grown under a condition of 16-h light and 8-h dark photoperiod, 28°C, and 80% soil humidity. When the cotyledons were fully open and the seedling height reached ~5 cm, the plantlets were pierced and inoculated with a drop of *A. rhizogenes* culture (OD_600_ = 0.6–0.8) at the hypocotyl position with a sterile syringe needle. The inoculated plantlets were covered with the transparent Saran film to keep humidity and were grown in the same condition as above. After a 15-day culture, the hairy roots emerged from the injection sites. The primary roots were cut off after the induced hairy roots reached 4–5 cm long. The plants with transgenic hairy roots were called composite plants. The healthy composite plants with a similar size and a hairy root number were selected for stress treatments. The physiological and phenotypic indexes, including biomass accumulation, shoot and root lengths, and chlorophyll contents, were determined after the plants were treated with 200 mM NaCl for 5–10 days.

### Total Protein Extraction and Protein Gel Blot Analysis

Total protein extraction from plants and protein gel blot analysis refer to the described method (Xiao et al., 2013). The histone-rich proteins were separated by 15% SDS-PAGE. Then, the proteins were transferred to the polyvinylidene fluoride (Biotrace TM, USA) membrane, blocked with 5% skimmed milk powder in TBST, and then treated with anti-H4ace [H4 hyperacetylation (K5K8K12K16)] (1:10,000; Millipore, anti-H4ace: 06598) to detect. Anti-H4 (1:10,000; Millipore, anti-H4: 07-108) was a loading control.

### Chromatin Immunoprecipitation Assays

A GFP-coding sequence was fused in frame to the 3′ end of the *GsMYST1* or *GsNAC83* gene, and the gene fusion was subcloned under the CaMV 35S promoter in the modified pCAMBIA3301 vector. Chromatin immunoprecipitation (ChIP) assays were performed as described (Bowler et al., 2004) with 14-day-old transgene hairy roots after being induced. Antibodies with specificity to H4ace (K5K8K12K16) were used to immunoprecipitate the chromatin (Xiao et al., 2013). The amount of immunoprecipitated *COR15B* chromatin was determined by real-time PCR on different regions of the *COR15B* locus. The primers are listed in Supplementary Table S1.

### Statistical Data Analysis

All experiments were carried out using biological duplications for each treatment and were replicated on at least three occasions. Multiple comparison tests were performed by Duncan's test using SPSS statistical software (version 16.0, SPSS). The significance level was set at *p* < 0.05.

## Results

### Identification of GsMYST1 From Wild Soybean

**Figure 1:**
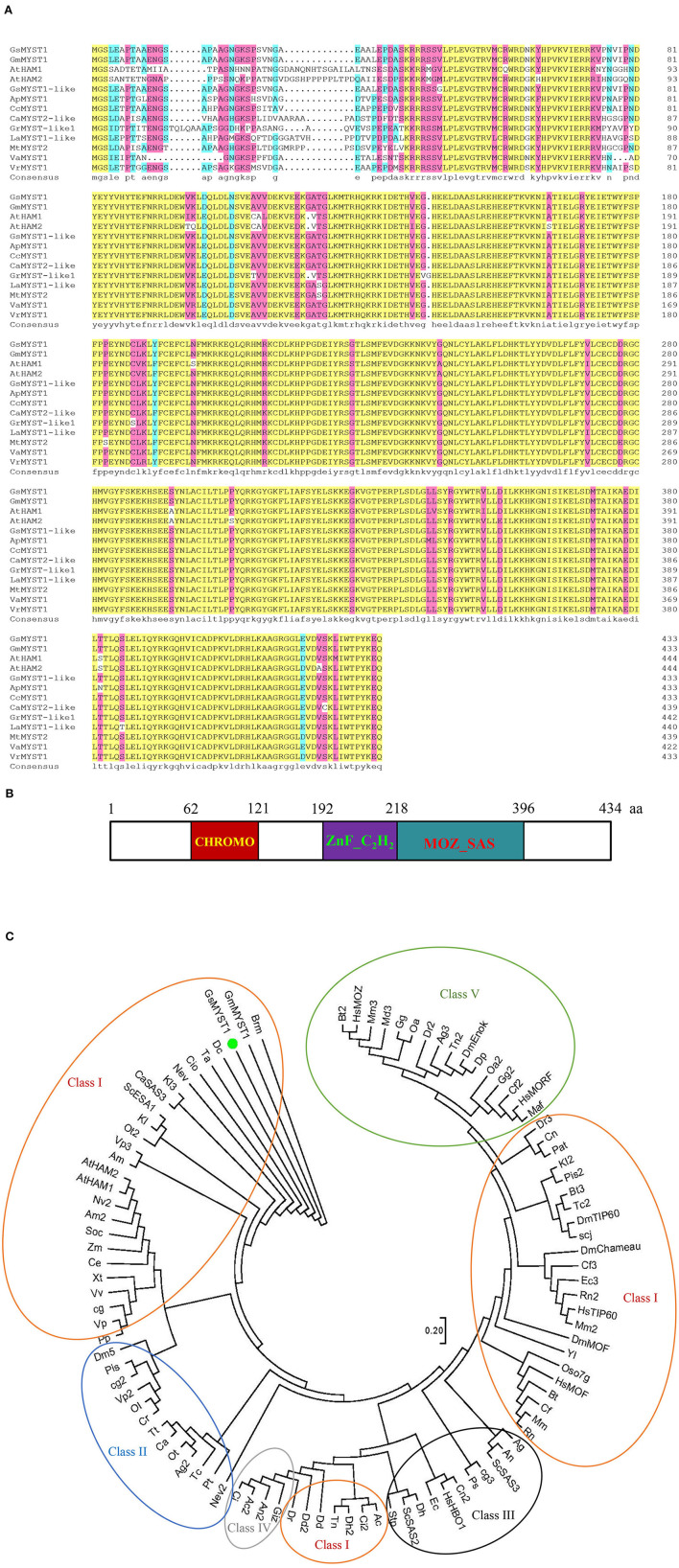
Sequence, structural, and phylogenetic analyses of GsMYST1. **(A)** The sequence alignment generated by DNAMAN. The protein sequences of the selected MYST family histone acetyltransferases were obtained from Genbank or Phytozome. The accession numbers are listed as follows: AtHAM1(At5g64610), AtHAM2(At5g09740), GsMYST1-like(XP_028228732.1), GmMYST1(XP_003522879.1), ApMYST1(XP_027341323.1), CcMYST1(XP_029129210.1), LaMYST1-like(XP_019431040.1), VrMYST1(XP_014499610.1), VaMYST1(XP_017425612.1), MtMYST2(XP_003598140.2), CaMYST2-like(XP_004499615.1), and GrMYST1-like1(XP_012451505.1). **(B)** Structural analysis of GsMYST1 protein. **(C)** Molecular phylogenetic analysis of the MYST family of histone acetyltransferase (HAT) taxa. The evolutionary history was inferred using the maximum likelihood method based on the JTT matrix-based model. The tree with the highest log likelihood (−71798.64) is shown. The initial tree for the heuristic search was obtained automatically by applying Neighbor-Join and BioNJ algorithms to a matrix of pairwise distances estimated using a JTT model and then selecting the topology with a superior log-likelihood value. The analysis includes 100 protein sequences. All positions containing gaps and missing data were eliminated. There are a total of 310 positions in the final dataset. Evolutionary analyses were conducted in MEGA7. Representations of color circles: red: Class I; blue: Class II; black: Class III; green: Class IV; orange: Class V.

A cDNA library in pGADT7-rec plasmid for Y2H screens was constructed using total mRNA from wild soybean (*G. soja*) seedlings (Song et al., 2019). GsSnRK1 (Glysoja.13G035870.1) in pGBKT7 was employed as bait to screen the obtained cDNA library, and a clone encoding an MYST1-like histone acetyltransferase (Glysoja.06G016367.1) (designated GsMYST1) was identified as the putative interactor of GsSnRK1. Sequence alignment and phylogenetic analyses indicate that GsMYST1 belongs to the MYST family of histone acetyltransferases. It has a peptide length of 434 amino acids and shares a high identity with *A. thaliana* MYST family histone acetyltransferase members AtHAM1 (At5g64610) and AtHAM2 (At5g09740) (Pandey et al., 2002). In Figure 1A, we can see that the C termini of various MYST1 proteins were highly conserved. Structural analysis using Pfam and SMART tools revealed that the MYST family proteins are highly similar. GsMYST1 possesses a chromodomain (PF 00385) in the N terminus and a zinc finger (C2H2 type) contiguous to a MOZ-SAS acetyltransferase domain (PF 01853) (Figure 1B). We further analyzed and compared the MYST family histone acetyltransferases from different species and constructed an evolutionary tree. The result indicates that the MYST family can be divided into five unrelated classes and wild soybean GsMYST1 as well as other homologs, including *Arabidopsis* HAM1 and HAM2, that belongs to class I (Figure 1C). *In silico* prediction shows that GsMYST1 protein has a significant nuclear localization signal (NLS) (Supplementary Figure S1A), and we experimentally proved that GsMYST1 protein is exclusively localized in the nucleus using GFP fusion protein (Supplementary Figure S1B).

### Expression of *GsMYST1* Gene in Wild Soybean

**Figure 2:**
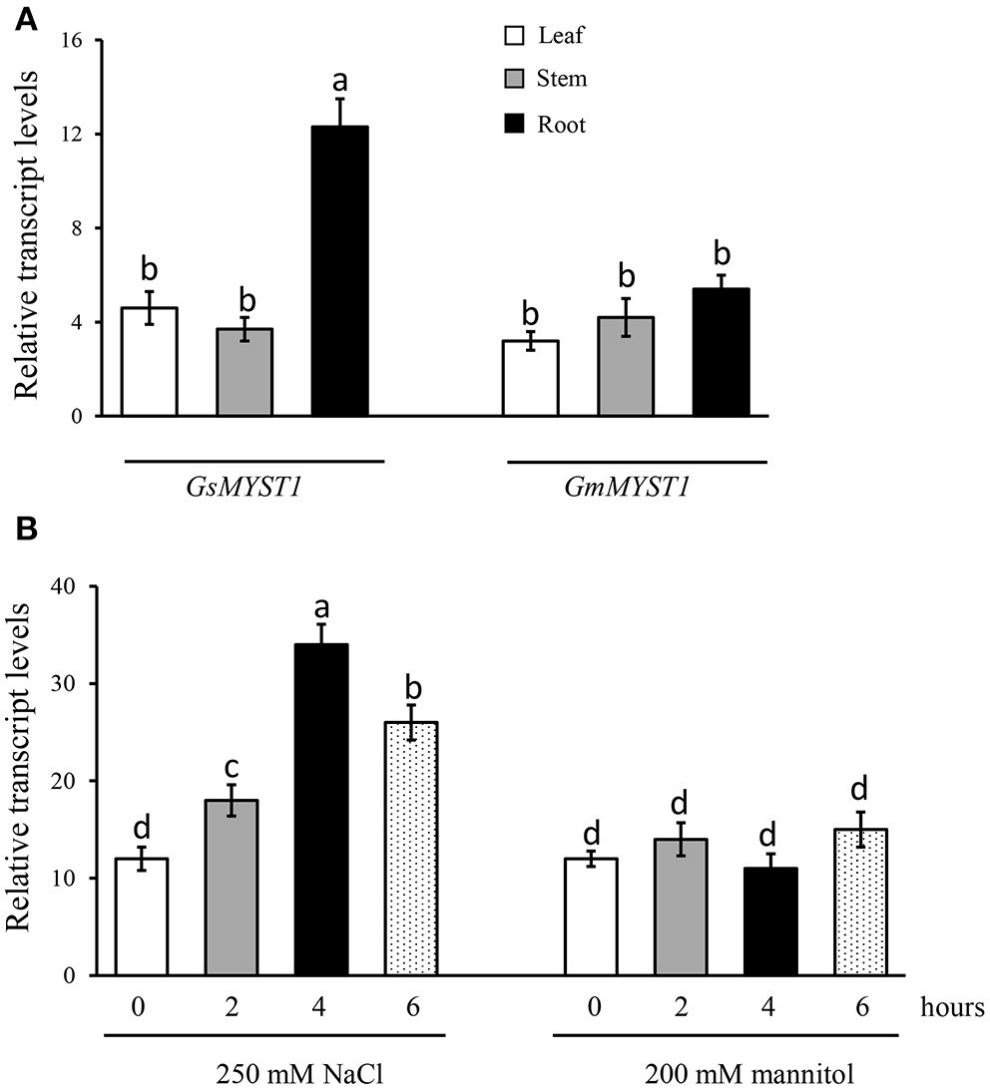
*GsMYST1* gene expression patterns in wild soybean. **(A)** The spatial expression patterns of *GsMYST1* and *GmMYST1* in different tissues of wild soybean and cultivated soybean were analyzed. Total RNA samples from the indicated tissues of 6-week-old adult plants were extracted for qRT-PCR. *GAPDH* was used as the internal reference. **(B)** Transcription levels of *GsMYST1* induced by abiotic stresses. The 6-week-old young wild soybean plants were treated with salt and mannitol for the indicated time periods, and then the total RNA samples were extracted from the treated plant roots for qRT-PCR analyses. Bars indicate SE, the t-test, and the columns were shown from three biological triplicates (*t*-test, *p* < 0.05, expressed in different small letter alphabets).

To investigate the spatial expression patterns and responses to environmental stimuli of the *GsMYST1* gene in wild soybean, we extracted total RNA from the plant different tissues and did qRT-PCR analyses. The results indicate that the *GsMYST1* gene is dominantly expressed in roots rather than in stems and leaves of wild soybean (Figure 2A). As a control, the *GmMYST1* gene was ubiquitously expressed in cultivated soybean at lower levels. Sequence comparison indicates that the promoters of *GsMYST1* and *GmMYST1* genes show high diversity and harbor different responsive elements (Supplementary Figure S2 and Supplementary Table S2). To determine if *GsMYST1* is responding to stresses in wild soybean, we treated wild soybean roots in 250 mM salt and 200 mM mannitol (dehydration) solutions for different time periods and analyzed *GsMYST1* expression levels. The qRT-PCR data suggested that the expressing level of *GsMYST1* in wild soybean roots was significantly induced by salt stress but not drought stress (Figure 2B). In contrast, we found that GmMYST1 in cultivated soybean did not show a response to abiotic stresses (Supplementary Figure S3). To confirm this observation, we cloned a 1.5-kb promoter sequence of *GsMYST1* and generated *GsMYST1*pro::*GUS* transgenic *Arabidopsis* plants. Treatment of the young transgenic *Arabidopsis* seedlings with 50 mM NaCl for 1 h significantly induced *GUS* expression in root and hypocotyl revealed by GUS staining (Supplementary Figure S4A) and quantitative β-glucuronidase enzymatic activity (Supplementary Figure S4B), suggesting that *GsMYST1* is a novel gene which may be involved in salt stress.

### Physical Interaction of GsSnRK1, GsMYST1, and GsNAC83

GsMYST1 was originally determined as an interactor of GsSnRK1 by the Y2H screen. As shown in Supplementary Figure S5, only the combination of GsMYST1/GsSnRK1 and the positive control but not the other combinations could promote yeast cell growth on the dropout medium lacking leucine, tryptophan, and histidine although all the yeast clones grew well on SD/-Leu-Trp medium. Moreover, the combination of GsMYST1 and GsSnRK1 in yeast cells could also activate the second reporter, the *MEL1* gene, which encodes secretory α-galactosidase enzyme to turn yeast colony color into blue on the medium containing X-α-gal. To explore the mechanism of how GsMYST1 functions on gene regulation, we further used GsMYST1 as a bait to screen wild soybean cDNA library using the Y2H approach and total identified 4 putative interactors of GsMYST1 (Supplementary Figures S6A,B). Considering the report of synergistic regulation of gene expression by kinase, acetyltransferase, and transcription factor in the plant (Poulios and Vlachonasios, 2016), we chose GsNAC83 transcription factor protein (Glysoja.05G012860.1) out of these interactors for further analyses. *GsNAC83* gene encodes a 241-aa polypeptide with a typical NAM/ATAF/CUC (NAC) domain on its N terminus (Supplementary Figure S7A). *In silico* prediction and subcellular localization analysis also show that GsNAC83 protein has significant NLS and is exclusively localized in the plant cell nucleus (Supplementary Figures S7B,C), suggesting that GsNAC83 may function as a transcription factor.

**Figure 3:**
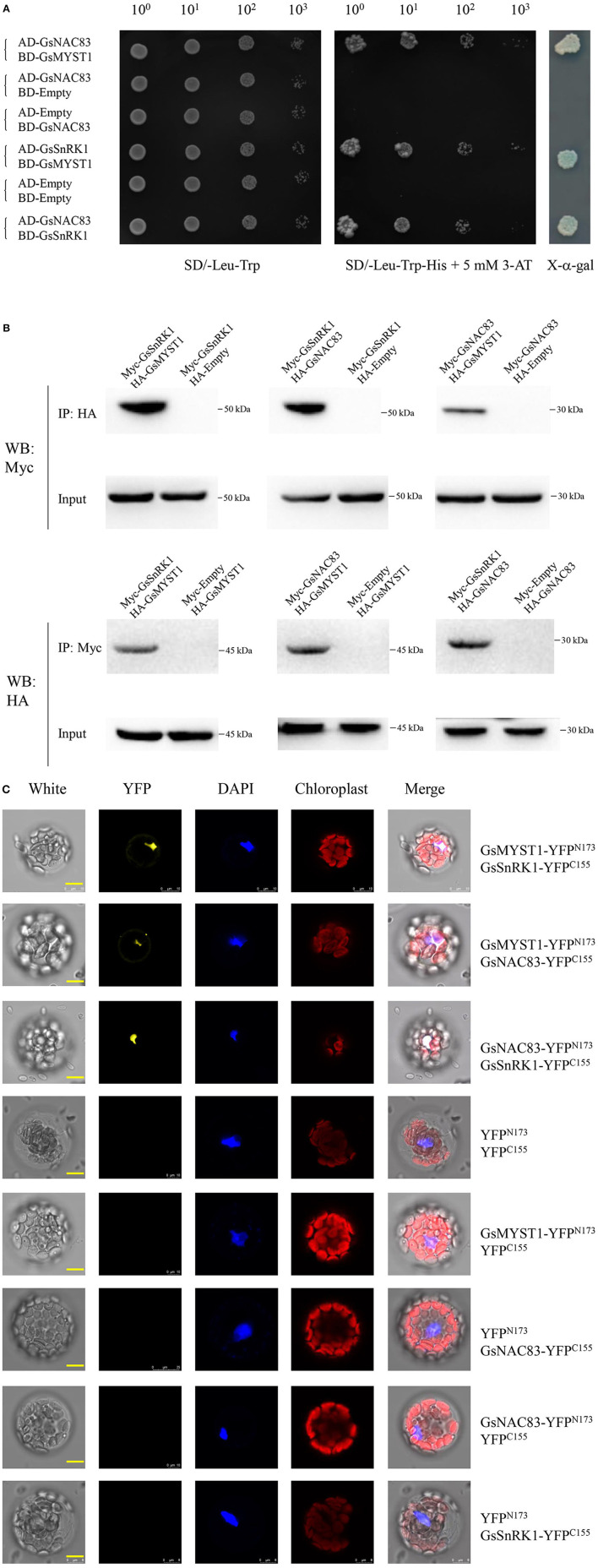
Physical interaction of GsSnRK1, GsMYST1, and GsNAC83. **(A)** Yeast two-hybrid assays: *GsSnRK1, GsMYST1*, and *GsNAC83* genes were cloned into AD or BD vectors. The resulted plasmids or the control vectors were cotransformed into Y2HGold yeast cells. The transformed clones were grown on the selective media with or without X-alpha-gal. **(B)** Co-immunoprecipitation assay: *GsMYST1, GsSnRK1*, and *GsNAC83* genes were cloned into vectors with HA or Myc tag. The obtained plasmids were coexpressed in *Arabidopsis* protoplasts. The total proteins were extracted for co-immunoprecipitation assays using the indicated antibodies. **(C)** BiFC assay: *GsMYST1, GsSnRK1*, and *GsNAC83* genes were cloned into the vectors with YFP^N173^ or YFP^C155^sequence. The resulted plasmids were transiently coexpressed in *Arabidopsis* protoplasts for 16 h. The YFP signals were observed under a confocal microscope. Each representative figure is shown from three biological triplicates.

NAC transcription factors belong to a plant-specific protein family playing significant roles in plant development and stress signaling (Jensen and Skriver, 2014). Given the respective interactions of GsMYST1 with GsSnRK1 and GsNAC83, it tempts us to investigate whether these three proteins associate with each other. First, we determined their interactions by Y2H. The results indicate that the pairwise combinations of these three genes could activate *HIS3* and *MEL1* reporter genes to make the yeast cells grow on SD-Leu-Trp-His medium and turn blue in the presence of X-α-gal (Figure 3A). Moreover, we transformed different pairs of plasmids into *Arabidopsis* protoplasts to transiently express the Myc/HA-tagged proteins and performed co-immunoprecipitation assays. As shown in Figure 3B, Myc-GsSnRK1 could be co-immunoprecipitated by HA-GsMYST1 and HA-GsNAC83, and Myc-GsNAC83 could be co-immunoprecipitated by HA-GsNAC83 (the upper panels). In an opposite way, we also co-immunoprecipitated the HA-tagged proteins by the Myc-tagged partners (the lower panels). To visualize where the proteins interact in the plant cells, we carried out BiFC assays in *Arabidopsis* protoplast cells. Consistent with the above observations, GsSnRK1, GsMYST1, and GsNAC83 can interact with each other by co-emitting yellow fluorescence in the plant cell nuclei (Figure 3C). Any individual proteins or empty plasmids did not generate a YFP signal, implicating that these three proteins specifically interact with each other *in vivo*. To further investigate whether these three proteins are co-localized in one protein complex, Myc-GsSnRK1, HA-GsMYST1, and Flag-GsNAC83 were cotransformed into *Arabidopsis* protoplasts to perform co-immunoprecipitation. The co-IP data showed that any two of the three proteins could be efficiently co-immunoprecipitated by the other partners, suggesting that GsSnRK1, GsMYST1, and GsNAC83 form a ternary protein complex in plant cells (Supplementary Figure S8).

### *In vitro* Phosphorylation of GsMYST1 by GsSnRK1

Considering the physical interaction among GsNAC83, GsMYST1, and GsSnRK1, it attracts us to investigate whether GsSnRK1 can phosphorylate GsMYST1 and GsNAC83. We used an online tool (http://ppsp.biocuckoo.org) to predict phosphorylation sites on these two proteins. The results showed that there was one potential SnRK1 phosphorylation site on GsMYST1(S44) with a risk-diff value of 6.32 and no phosphorylation site on GsNAC83 (Supplementary Tables S3, S4). To confirm this prediction, we expressed and purified the recombinant proteins of wild-type GsMYST1, GsNAC83, GsSnRK1, and their mutants in *Escherichia coli*. The phosphorylation reactants were fractionated by 12% SDS-PAGE gel containing 20 μM phos-tag reagent and 100 μM Mn^2+^, which is widely used in the evaluation of protein phosphorylation. To accurately pinpoint the phosphorylation site of GsMYST1 by GsSnRK1, we generated the GsMYST1(S44A) mutant at the same time.

**Figure 4:**
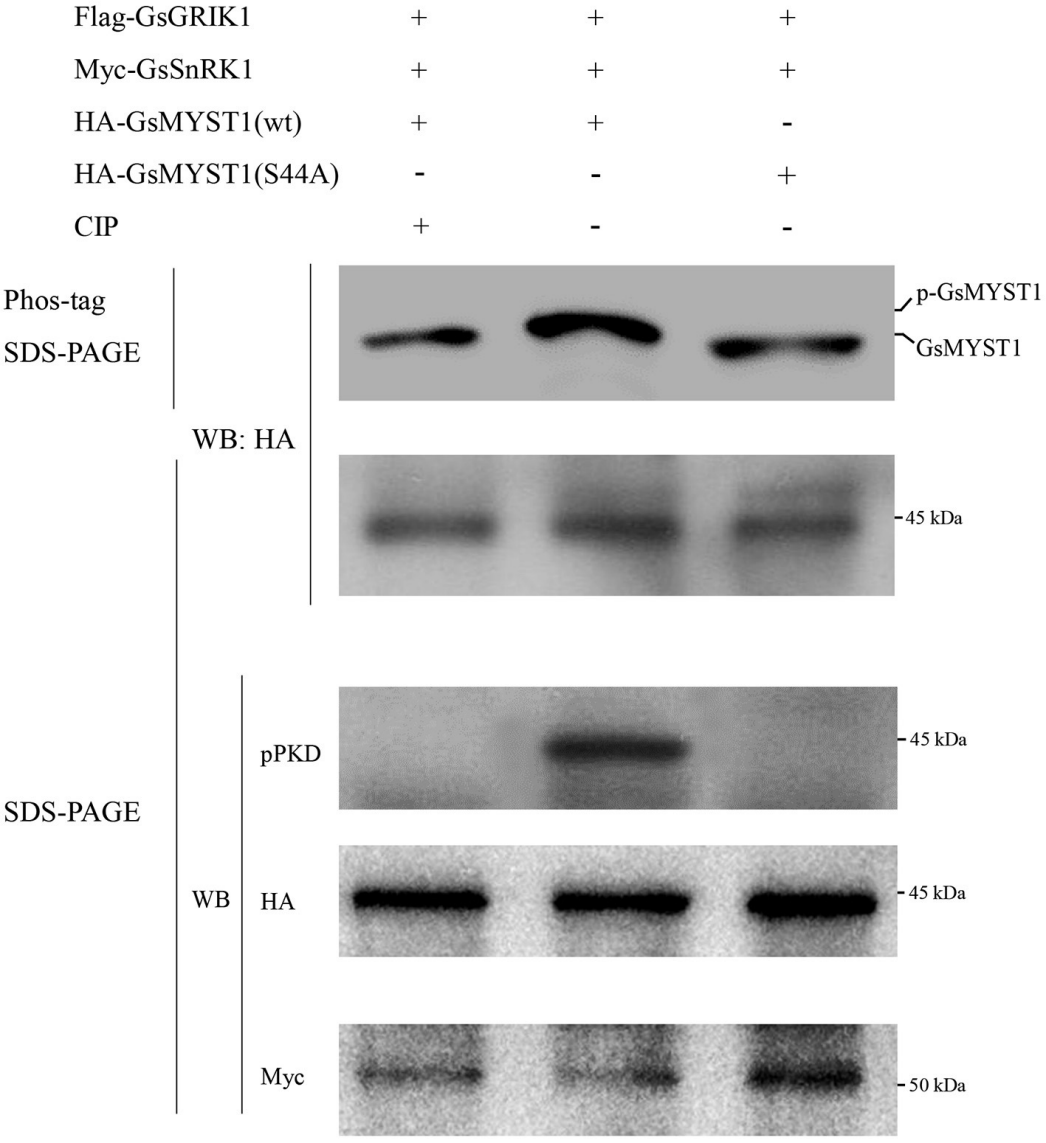
*In vitro* GsMYST1 phosphorylation assay. GsSnRK1(wt), GsMYST1(wt), and their mutants were expressed and purified in *E. coli*. The indicated proteins were combined and incubated for kinase reactions. The reactants were fractionated in SDS-PAGE gels with or without Phos-tag. The membranes were, respectively, blotted with the indicated antibodies to show the protein phosphorylation status and loading controls. Each representative figure is shown from three biological triplicates.

After the recombinant proteins were incubated for phosphorylation reactions, the reactants were fractionated in PAGE gel with Phos-tag. WB data showed that in the presence of GsSnRK1(wt) and its upstream kinase GsGRIK1, GsMYST1(wt) exhibited an obvious upshifted protein band but this upshifted GsMYST1(wt) protein species could be wiped off by treatment of calf intestinal alkaline phosphatase (CIP). However, when the mutant GsMYST1(S44A) was incubated with GsSnRK1, GsMYST1(S44A) did not show any upshifted protein band (the upper two panels in Figure 4). In addition, we used the phosphor-(Ser/Thr) PKD substrate antibody to recognize the phosphorylated Ser44 of GsMYST1. Consistent with the above results, we detected a specific phosphorylation band from the combination of GsSnRK1(wt)/GsMYST1(wt) using the phosphor-(Ser/Thr) PKD substrate antibody. The same as above, CIP treatment could effectively eliminate the phosphorylated band, and GsMYST1(S44A) also did not demonstrate any phosphorylation signal (the middle panel in Figure 4), indicating that GsMYST1 is the *bona fide* phosphorylation substrate of GsSnRK1, and Ser44 in GsMYST1 is a unique phosphorylation site by GsSnRK1. Nevertheless, we also measured the phosphorylation status of GsNAC83 by GsSnRK1 but did not find any phosphorylation signal of GsNAC83 (data not shown).

### *In vivo* Phosphorylation of GsMYST1 by GsSnRK1 in Soybean Roots

**Figure 5:**
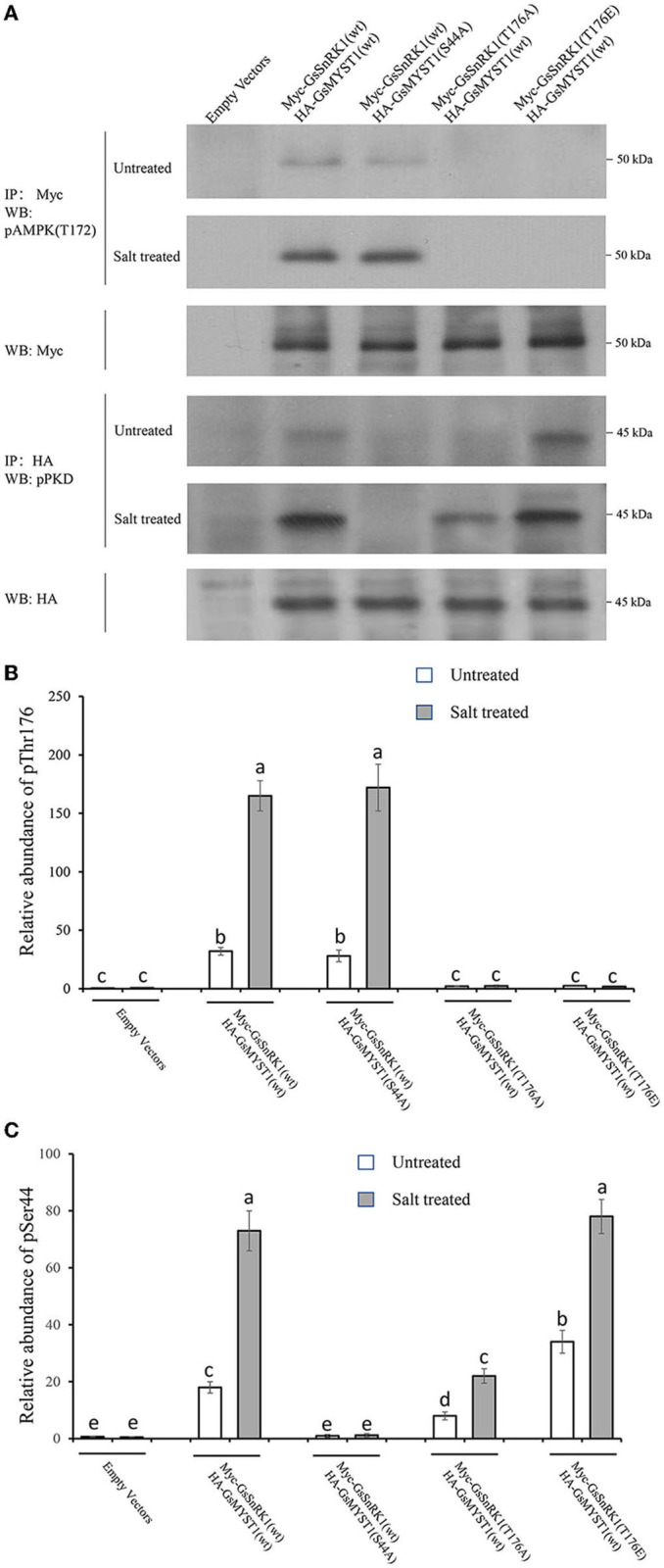
*In vivo* GsMYST1 phosphorylation assays. **(A)** The indicated plasmids were cotransformed into soybean hairy roots mediated by *Agrobacterium rhizogenes*. The total proteins were extracted from the roots after the plants were treated with 150 mM NaCl for 2 h. Myc-GsSnRK1 or HA-GsMYST1 were immunoprecipitated from total proteins using mouse anti-Myc or anti-HA antibodies. The immunoprecipitated proteins were resolved in SDS-PAGE gels for WB using the indicated antibodies. **(B)** The relative phosphorylation levels of Thr176 in GsSnRK1 before and after salt treatment. **(C)** The relative phosphorylation levels of Ser44 in GsMYST1 before and after salt treatment. Bars indicate SE, the t-test, and the columns were shown from three biological triplicates (*t*-test, *p* < 0.05, expressed in different small letter alphabets).

Although GsMYST1 was determined to be the phosphorylation substrate of GsSnRK1 *in vitro*, we need to ascertain whether and how GsSnRK1 phosphorylates GsMYST1 *in planta*. First, we cotransformed GsSnRK1(wt)/GsMYST1(wt) and their mutants into soybean hairy roots and extracted total proteins. WB using anti-HA and anti-Myc antibodies showed that HA-GsMYST1(wt)/Myc-GsSnRK1(wt) and their mutants were all properly expressed (Supplementary Figure S9). Second, we treated the transgenic soybean plants with 150 mM NaCl for 2 h and then extracted proteins from the roots for protein phosphorylation assays. The Myc-GsSnRK1 and HA-GsMYST1 proteins were immunoprecipitated for WB using phospho-AMPK(Thr172) antibody and phospho-PKD substrate antibody. Since Thr210 of yeast SNF1, Thr172 of mammalian AMPK kinase, and Thr176 of wild soybean GsSnRK1 are functionally conserved. Phosphorylation of these sites by their upstream kinases can activate SNF1/AMPK/SnRK1 kinases. Intriguingly, our WB data indicated that the signals of phospho-Thr176 of GsSnRK1(wt) were greatly enhanced after salt treatment revealed using the phospho-AMPK(Thr172) antibody, but mutation of T176A and T176E abrogated the signal, suggesting that phosphorylation of this site is highly specifically responsive to salt stress (the upper two panels in Figure 5A) (Figure 5B). WB data using the anti-phospho-PKD substrate antibody showed that GsMYST1 was able to be phosphorylated at basal levels to different extents under the favorite condition but salt stress could significantly enhance phosphorylation of GsMYST1 (the 4th and 5th panels in Figure 5A) (Figure 5C). Phosphomimic mutant GsSnRK1(T176E) could generate a stronger phosphorylation signal of GsMYST1 than GsSnRK1(wt). The phosphorylation signal of GsMYST1 in the presence of GsSnRK1(T176A) might have resulted from endogenous kinases.

### Enzymatic Activity of Histone Acetyltransferase GsMYST1

In *A. thaliana*, HAM1 and HAM2, members of the MYST family, have the enzymatic function of acetylated histone H4 (Xiao et al., 2013). Due to their functional conservation in evolution, it is speculated that GsMYST1 also has the function of acetylated histone H4 in plants. To aim this, we overexpressed the *GsMYST1* gene and silenced the *GmMYST1* gene by RNAi in soybean hairy root. We first examined the expression levels of the *GsMYST1* gene and *GmMYST1* gene in overexpression lines and RNAi lines by qRT-PCR analyses. As a result, we detected high-level expression of *GsMYST1* and repressed expression of *GmMYST1* genes in the overexpression and RNAi lines, respectively (Supplementary Figures S10A,B), and we also used an anti-GFP antibody to confirm the expression of GFP-GsMYST1 in the transgenic soybean roots (Supplementary Figure S11). Then, we employed an anti-histone H4 hyperacetylation (K5K8K12K16) antibody to detect the acetylation level of histone H4 in *GFP*-OE, *GFP-GsMYST1*-OE, and *GmMYST1*-RNAi lines. The WB data suggested that the GFP-GsMYST1 line has the highest acetylation level of histone H4, whereas the RNAi-GmMYST1 line has the lowest acetylation level of histone H4 (Supplementary Figures S12A,B), implicating that GsMYST1 plays an active role of histone acetyltransferase *in vivo*.

### Transcription Factor GsNAC83 Regulates the Expression of the *COR15B* Gene

**Figure 6:**
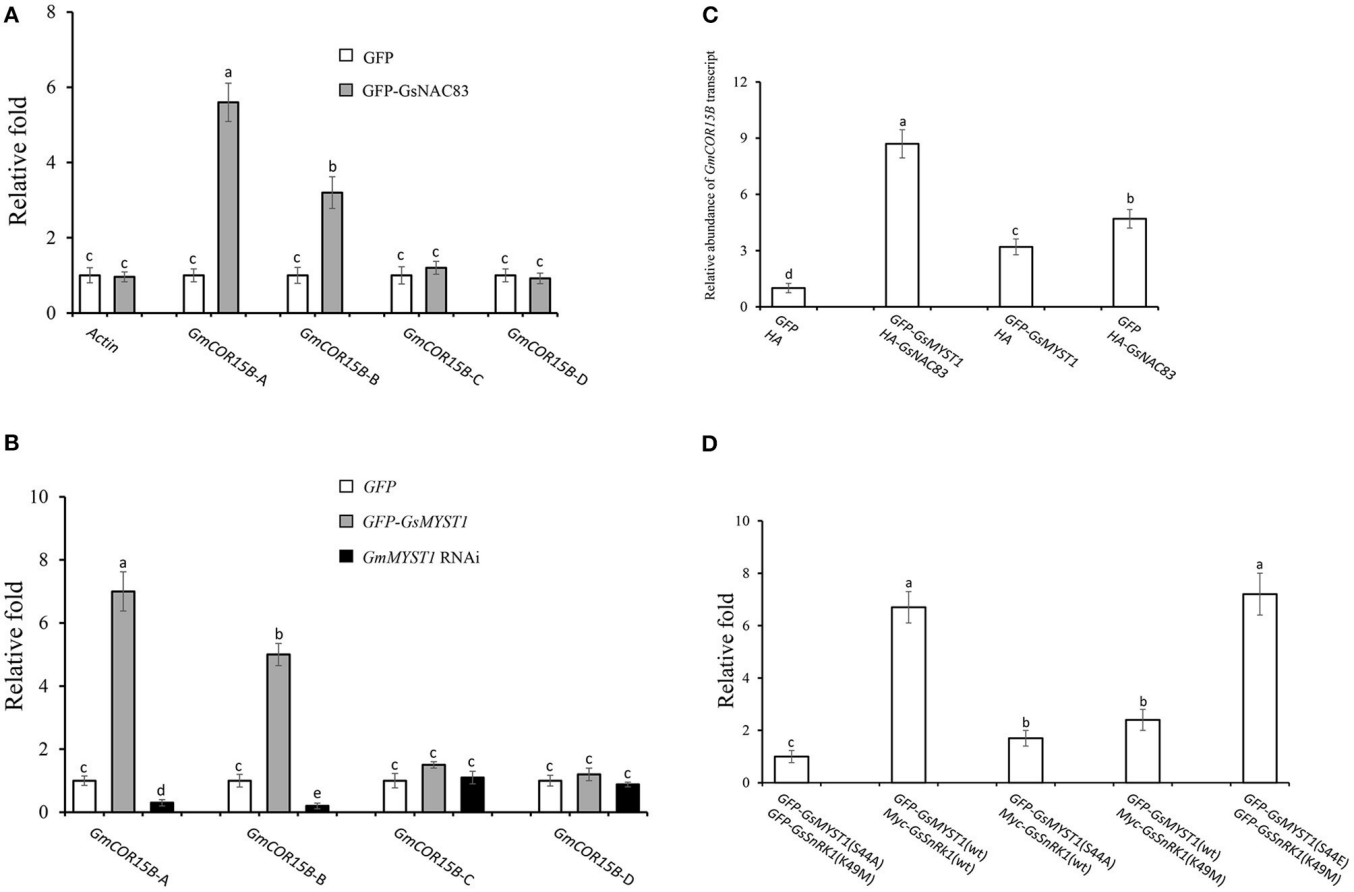
Regulation of *COR15B* gene expression by GsSnRK1-GsMYST1-GsNAC83G module. **(A)** Analyses of binding sites of GsNAC83 on *COR15B* promoter using ChIP-PCR approach. **(B)** Analyses of acetylation of *COR15B* gene by GsMYST1 using ChIP-PCR approach. **(C)** Effect of GsNAC83 and GsMYST1 on the expression of *COR15B* revealed using qRT-PCR. **(D)** Effect of GsMYST1 phosphorylation by GsSnRK1 on acetylation of *COR15B* gene. Bars indicate SE, the t-test, and the columns were shown from three biological triplicates (*t*-test, *p* < 0.05, expressed in different small letter alphabets).

Histone acetyltransferase usually forms complexes with other transcription factors to regulate the expression of downstream target genes (Poulios and Vlachonasios, 2016). Since GsSnRK1 and GsMYST1 have no DNA-binding domain, GsSnRK1 and GsSMYST1 are supposed to be recruited to certain DNA sequences by GsNAC83. We have already determined that GsSnRK1, GsMYST1, and GsNAC83 can form heterotrimeric complexes. In *Arabidopsis*, AtNAC83 is also known as AtVNI2 (At5g13180). Sequence alignment indicates that GsNAC83 has a high identity with AtVNI2 (Supplementary Figure S13). It has been reported that AtVNI2 targets *COR* and *RD* family gene members, namely, *COR15A, COR15B, RD29A*, and *RD29B* to integrate abscisic acid signals into tolerance to abiotic stresses (Yang et al., 2011), which attracts us to investigate whether GsNAC83 also targets these stress-related genes to participate in the regulation of stress resistance in soybean. We surveyed the promoter sequences of *COR15A, COR15B, RD29A*, and *RD29B* genes in the soybean genome and found that only the *GmCOR15B* promoter has a conserved sequence motif which is analogous to the consensus sequences (CATGT) for binding of NAC transcription factors (Olsen et al., 2005). Thus, we overexpressed GFP-GsNAC83 or GFP in soybean hairy roots (Supplementary Figure S14) and used the chromatin immunoprecipitation quantitative PCR (ChIP-qPCR) approach to determine where GsNAC83 binds in the promoter of *GmCOR15B* gene. First, we performed ChIP using an anti-GFP antibody and then did qPCR analyses using the primer pairs located in the promoter, 5′ UTR, intron, and exon (Supplementary Figure S15). The ChIP-qPCR data indicated that the enrichment rate of the *COR15B-*A region was significantly higher than those of the other regions, implicating that GsNAC83 may specifically bind to *GmCOR15B* promoter and 5′ UTR (Figure 6A). To confirm the results, we synthesized a GsNAC83 binding sequence (BS: TAGATA**CATGT**TGCA) as bait to do Y1H. The result showed that GsNAC83 could specifically bind the BS sequence but not bind the mutated mBS sequence (TAGAT**CCCCC**TTGCA) (Supplementary Figure S16).

### GsNAC83 Mediates GsMYST1 to Acetylate the Promoter of the *GmCOR15B* Gene

Given the interaction of GsNAC83 with GsMYST1, we need to understand if GsMYST1 participates in regulating *GmCOR15B* expression. Based on Figure 6A data, we carried out ChIP-PCR assays using anti-histone H4 hyperacetyl antibody in *GFP, GFP-GsMYST1*, and *GmMYST1* RNAi transgenic soybean hairy roots. The qPCR results showed that histone H4 hyperacetylation was highly enriched in both promoter (*GsCOR15B*-A) and 5′ UTR (*GsCOR15B*-B) but not in the intron and exon of the *GmCOR15B* gene (Figure 6B), consistent with GsNAC83 DNA binding data (Figure 6A). We further analyzed *GmCOR15B* expression in different transgenic soybean roots by qRT-PCR, and the results showed that the expression of the *GmCOR15B* gene is largely dependent on the presence of both GmMYST1 and GsNAC83 (Figure 6C), suggesting that *GsCOR15B* promoter region is highly possible to be acetylated by GsMYST1 mediated by GsNAC83, which results in upregulation of *GsCOR15B* expression.

### Phosphorylation of GsMYST1 by GsSnRK1 Is Required for Acetylation of Histone H4 in *GmCOR15B*

To explore whether the phosphorylation of GsMYST1 affects its acetyltransferase activity, we coexpressed *GsSnRK1*(wt) or *GsSnRK1*(K49M) with *GFP*-*GsMYST1*(wt) or *GFP*-*GsMYST1*(S44A) in soybean hairy roots and performed ChIP assay using anti-histone H4 hyperacetyl antibody. The ChIP-qPCR results showed that the acetylation level of histone H4 in the promoter region of *COR15B* was compromised in the presence of GsMYST1(S44A) or GsSnRK1(K49M) but the acetylation level of histone H4 in the promoter region of *COR15B* was enhanced in the presence of GsMYST1(wt)/GsSnRK1(wt) and phosphomimic mutant GsMYST1(S44E) (Figure 6D), implicating that phosphorylation of GsMYST1 by GsSnRK1 is required for acetylation of histone H4 in *GmCOR15B* promoter.

### Salt Stress Promotes *GmCOR15B* Expression *via* the GsSnRK1-GsMYST1-GsNAC83 Module

**Figure 7:**
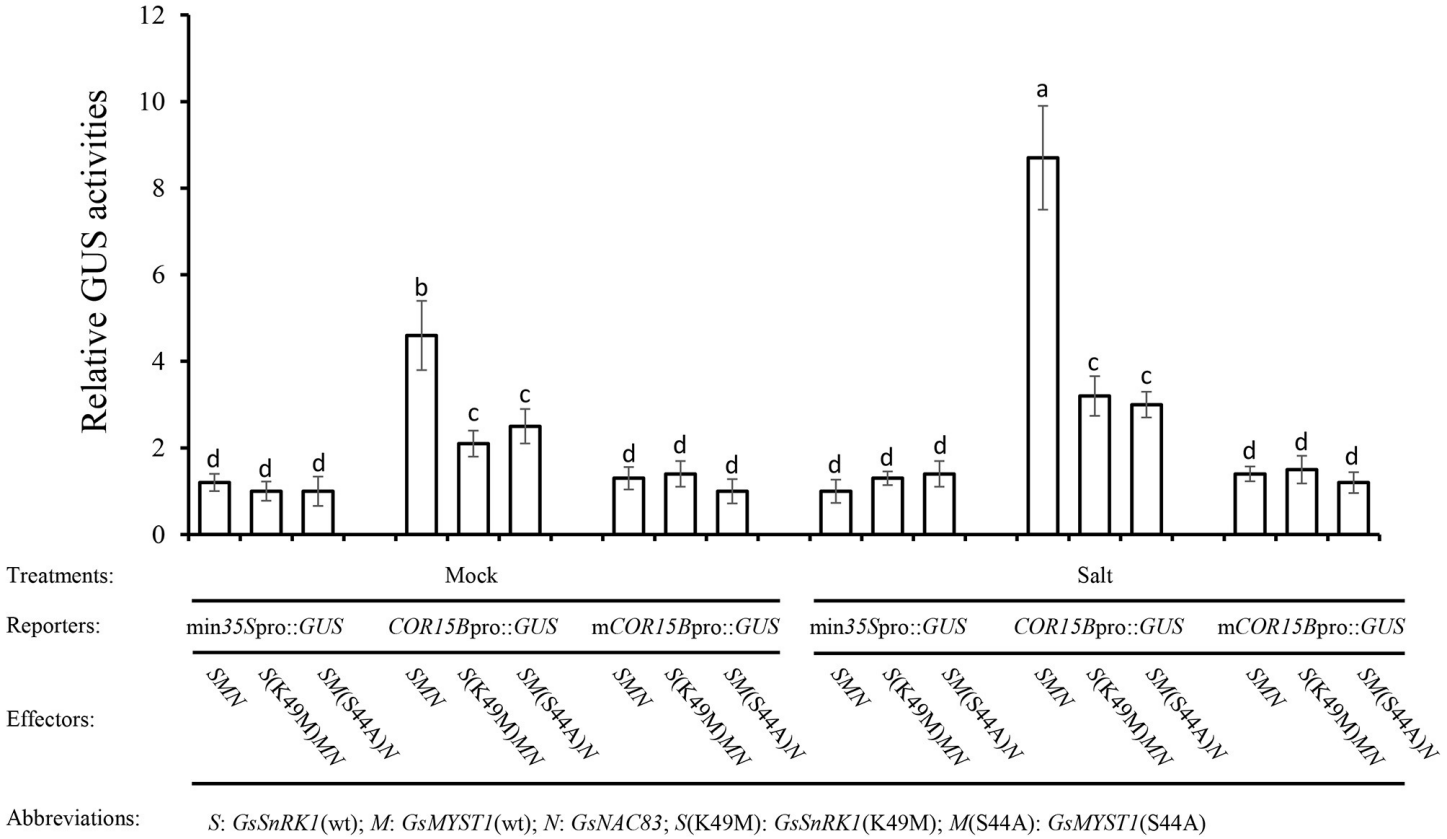
GsSnRK1-GsMYST1-GsNAC83G module mediates salt stress signal. The reporter and the effector constructs were cotransformed into *Arabidopsis* protoplasts. After 16-h incubation, the protoplasts were treated with 10 mM NaCl for 2 h, and total proteins were extracted for quantitative measurements of GUS activities. Bars indicate SE, the t-test, and the columns were shown from three biological triplicates (*t*-test, *p* < 0.05, expressed in different small letter alphabets).

To explore the molecular mechanism of how the salt stress signal is transduced through the GsSnRK1-GsMYST1-GsNAC83 module to stimulate the expression of salt-resistant genes, we generated a series of effector and reporter constructs (Supplementary Figure S17). The reporter construct (min*35S*pro::*GUS, COR15B*pro::*GUS*, or the mutated m*COR15B*pro::*GUS*) and the indicated effector constructs were cotransformed into *Arabidopsis* protoplasts. A vector construct containing the luciferase (LUC) gene was included to monitor transformation efficiency. After incubation for 16 h, the protoplasts were treated with 10 mM NaCl for 2 h, and then, the total proteins were extracted for quantitative measurement of GUS activities. Under mock and salt stress conditions, the GUS activities from the *COR15B*pro::*GUS* group were significantly higher than those from min*35S*pro::*GUS* and m*COR15B*pro::*GUS* groups, suggesting that *COR15B*pro::*GUS* can be specifically activated by the GsSnRK1-GsMYST1-GsNAC83 module. Since the promoter sequences of *COR15B* genes in cultivated and wild soybean are almost identical (Supplementary Figure S18), this signaling pathway may be conserved in these two species. It is intriguing that salt stress tremendously enhanced GUS activity from the protoplasts transformed with *COR15B*pro::*GUS*, GsSnRK1(wt), GsMYST1(wt), and GsNAC83. Noticeably, when kinase-dead mutant [GsSnRK1(K49M)] or GsMYST1(S44A) presented, the GUS activity was apparently abrogated (Figure 7). These results further prove that activation of the stress-related *COR15B* gene is mediated by the GsSnRK1-GsMYST1-GsNAC83 module, which is highly consistent with the above biochemical and molecular data.

### GsSnRK1 and GsMYST1 Promote Soybean Resistance to Abiotic Stress

**Figure 8:**
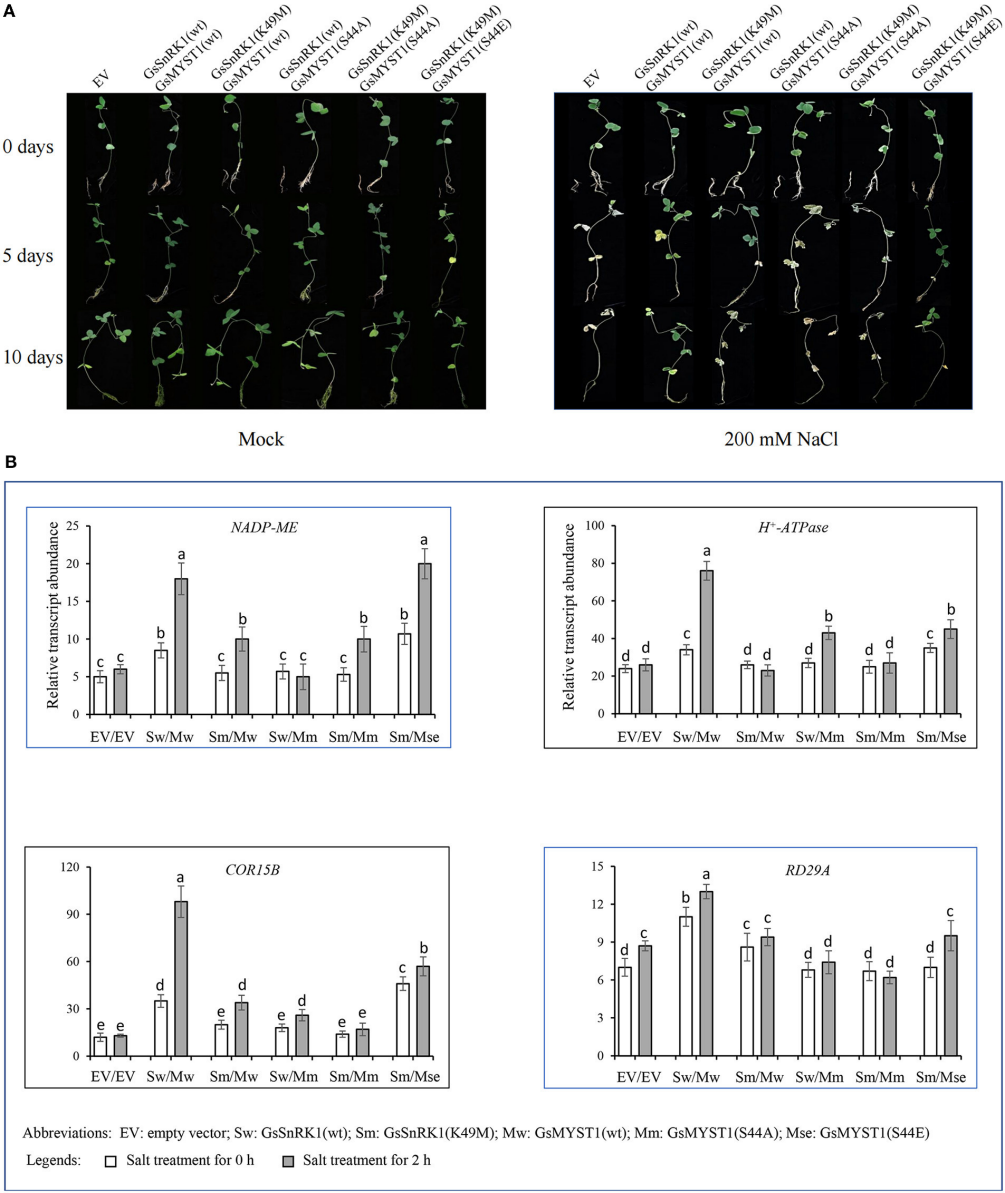
GsSnRK1 and GsMYST1 enhance soybean resistance to salt stress. **(A)** *GsSnRK1*(wt), *GsMYST1*(wt), and their mutants were cotransformed into soybean hairy roots followed by treatment of 200 mM for 5–10 days. The phenotypes of representative plants were observed and compared. Each representative figure was shown from three biological triplicates. **(B)** The expression patterns of four stress-related genes from soybean hairy roots cotransformed with the indicated genes. Bars indicate SE, the t-test, and the columns were shown from three biological triplicates (*t*-test, *p* < 0.05, expressed in different small letter alphabets).

To study the physiological function and genetic relationship of GsSnRK1 and GsMYST1, we overexpressed *GsSnRK1* and *GsMYST1* genes and their mutants in soybean hairy roots. When growing in a normal medium, all soybean plants with different genotypes of roots demonstrated similar growth, biomass, chlorophyll content, and shoot and root length. However, after treatment with 200 mM NaCl for 5–10 days, the soybean plants exhibited different phenotypes. The plants transformed with *GsSnRK1*(wt)/*GsMYST1*(wt) as well as *GsSnRK1*(K49M)/*GsMYST1*(S44E) exhibited much more pronounced growth than the other lines, further proving that phosphorylation at Ser44 of GsMYST1 is critical for its acetyltransferase activity and positive regulation of salt resistance. The other lines [empty vector, *GsSnRK1*(K49M)/*GsMYST1*(wt), *GsSnRK1*(wt)/*GsMYST1*(S44A), and *GsSnRK1*(K49M)/*GsMYST1*(S44A)] showed apparently arrested growth with wilting and chlorotic leaves and bald roots with few lateral and hairy roots (Figure 8A). These data suggest that the overexpressed wild soybean GsSnRK1 and GsMYST1 may interact with endogenous GmNAC83 to synergistically regulate soybean resistance to salt stress.

To investigate how the GsSnRK1-GsMYST1-GsNAC83 module regulates soybean gene expression on salt stress, we then measured the expression patterns of four representative stress-related marker genes (*NADP-ME, H*^+^*-ATPase, COR15B*, and *RD29A*) in the different transgenic soybean hairy roots treated with 200 mM NaCl for 2 h. The qRT-PCR data indicated that the expressions of marker genes were significantly enhanced in the transgenic soybean lines with *GsSnRK1*(wt)/*GsMYST1*(wt) and *GsSnRK1*(K49M)/*GsMYST1*(S44E) compared with the other lines (Figure 8B). These four marker genes may be directly or indirectly regulated by the GsSnRK1-GsMYST1-GsNAC83 module.

## Discussion

SnRK1 kinases function in crucial roles in signaling transduction involved in plant growth and stress responses. So far, the molecular mechanisms regulating SnRK1 complex function are still largely unknown, and only few substrates have been identified. This is in contrast to 216 physical interactors identified for SNF1 in yeast and over 60 targets identified through phosphorylation prediction and high-throughput studies for the mammalian AMPK (Emanuelle et al., 2016), pointing to a need for large scale identification of SnRK1 interactors in plants. Activation of SnRK1 or stress treatment results in 20–30% overlapping transcriptional changes, suggesting that these important stress pathways share common target genes. In this study, we used the Y2H approach to uncover SnRK1 interactors. One of these interactors is an acetyltransferase GsMYST1 from wild soybean. Till now, no report about how SnRK1 regulates any acetyltransferases to perform epigenetic regulation of plant gene expression.

**Figure 9:**
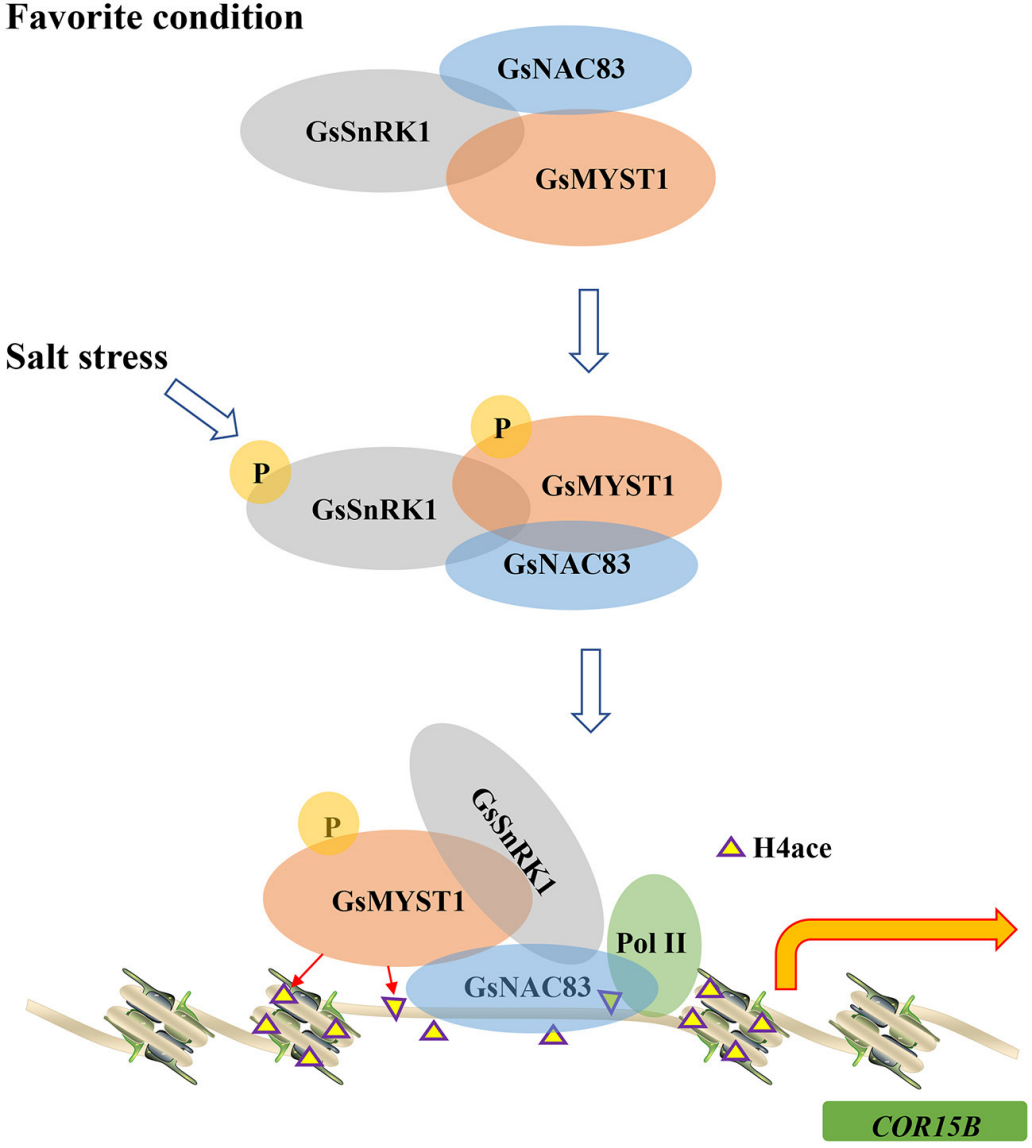
A model of GsSnRK1-GsMYST1-GsNAC83 regulating soybean response to salt stress. Under the favorite condition, GsSnRK1, GsMYST1, and GsNAC83 interact with each other to form a ternary complex. On salt stress from the environment, the upstream kinase (e.g., GRIK1) phosphorylates Thr176 to activate GsSnRK1, and the activated GsSnRK1 phosphorylates Ser44 of GsMYST1. The phosphorylated GsSnRK1 and GsMYST1 are recruited to the *COR15B* gene promoter by the GsNAC83 transcription factor. GsMYST1 acetylates histone H4 to stimulate expression of the *COR15B* gene to enhance plant resistance to salt stress.

Histone posttranslational modifications play key roles in modulating chromatin structure and gene transcription. The acetylation of core histones usually induces an “open” chromatin structure that associates with gene activation, whereas deacetylation of the histone induces a “closed”' chromatin structure and gene repression (Liu et al., 2014). The results from this study allow us to formulate a molecular model that integrates the role of histone acetylation into the transcriptional regulation of the *COR15B* gene. In this study, we found that GsSnRK1, GsMYST1, and GsNAC83 interact with each other to form a heterotrimeric complex. Since GsSnRK1 and GsMYST1 have no DNA binding domain, we proposed a model that GsSnRK1 and GsMYST1 should be recruited to the target genes through the NAC83 transcription factor (Figure 9). Through chromatin immunoprecipitation PCR (ChIP-PCR) assays, we identified a binding site in the promoter of the *COR15B* gene. We further determined that GsSnRK1 phosphorylates GsMYST1 when the plants are subject to salt stress, and phosphorylated GsMYST1 can play as a histone acetyltransferase to acetylate the transcription initiation region of *COR15B*, the downstream target gene of transcription factor GsNAC83. Histone acetylation is thought to release tight chromatin at the beginning of transcription to facilitate the entry of RNA polymerase complexes (Kouzarides, 2007). Similar results were reported about the synergistic action of GCN5 acetyltransferase and CLV1 kinase in the regulation of gynoecium development in *A. thaliana*. Both GCN5 and CLV1 regulate inflorescence meristem size and affect the expression of the meristem-promoting transcription factor WUSCHEL (WUS). Although it is still unclear whether GCN5 and CLV1 biochemically regulate each other, CLV1 and GCN5 can affect histone H3 acetylation in *WUS* and auxin-related genes. In the double mutants, all the loci tested displayed reduced total H3 and H3K9/14 acetylation levels, indicating that CLV1 and GCN5 act synergistically as positive regulators of histone acetylation (Poulios and Vlachonasios, 2016).

In our results, we detected significant acetylation signals using an anti-histone H4 hyperacetylation (K5K8K12K16ace) antibody in a GsNAC83 target gene, *COR15B*, and these acetylation signals were largely dependent on GsMYST1 phosphorylation by GsSnRK1. Phosphorylation is a kind of protein modification, which can significantly alter protein conformations and functions. In our previous study, we found that GsSnRK1 can interact with and phosphorylate transcription factor GsERF7. Phosphorylation of GsERF7 changed its subcellular localization and enhanced its transcription ability and thus promoting plant resistance to salt-alkaline stresses (Feng et al., 2020).

In this study, we also identified a transcription factor GsNAC83, but it is not a phosphorylation substrate of GsSnRK1. Many transcription factors act as either transcriptional inhibitors or activators, depending on the cellular environment. Some NAC transcription factors may contain transcriptional activation and repression domains. In our case, although GsNAC83 can activate *COR15B* gene expression to a certain level, cotransformation of GsSnRK1 and GsMYST1 tremendously enhanced the target gene expression (Figure 7), implicating that GsNAC83 may function as a bridge to recruit GsMYST1 and GsSnRK1 to epigenetically upregulate the target gene expression. It is necessary to clarify this observation using the genetic approach.

The *COR* genes are regulated by both ABA-dependent and ABA-independent stress signals. Promoters of these genes contain conserved sequence motifs, such as CRT/DRE (TACCGACAT) and ABA-responsive elements (PyACGTGGC). The *cis*-acting elements serve as nodes in molecular webs that integrate incoming signals. As inferred from the presence of a series of distinct *cis*-acting elements in the gene promoters, the *COR* genes are influenced by various developmental and environmental cues, such as ABA, dehydration, and cold, supporting the idea that the *cis*-acting elements are one of the major sites for signaling crosstalk. Previous studies have found that COR proteins play an important role in AtVNI2-mediated development and stress signal integration during leaf senescence under salt stress (Yang et al., 2011). Leaf aging and osmotic stress can cause damage to chloroplast and other organelles and cell proteins. After treating *GsMYST1* transgenic soybean hairy roots with salt stress, we also found significant changes in senescence-related marker genes, especially *COR* family genes (Figure 8B).

In all, we identified a GsSnRK1-GsMYST1-GsNAC83 module from wild soybean which can mediate epigenetic regulation of *COR* gene to contribute resistance to salt stress in soybean, providing a clue to uncover novel molecular mechanisms of plant adaptabilities to harsh environments.

## Data Availability Statement

The original contributions presented in the study are included in the article/Supplementary Materials, further inquiries can be directed to the corresponding author/s.

## Author Contributions

PF, XD, and YD designed the project. XS and XL did soybean and *Arabidopsis* transformation and WB. YL, QS, and HL did gene cloning, Y1H, and Y2H. ML and YL did BiFC and co-IP. PF and XD wrote the manuscript. All authors contributed to the article and approved the submitted version.

## Funding

This study was financially supported by the Agricultural Science and Technology Innovation Project of Jilin Province (CXGC2018ZY010) to XL, the National Natural Science Foundation of China (31901566) to YL, the National Key R&D Plan (2021YFD1201104-02) to XD, and Heilongjiang Academy of Agricultural Sciences (2021YYYF006) to XS.

## Conflict of Interest

The authors declare that the research was conducted in the absence of any commercial or financial relationships that could be construed as a potential conflict of interest.

## Publisher's Note

All claims expressed in this article are solely those of the authors and do not necessarily represent those of their affiliated organizations, or those of the publisher, the editors and the reviewers. Any product that may be evaluated in this article, or claim that may be made by its manufacturer, is not guaranteed or endorsed by the publisher.

## Abbreviations

SnRK: sucrose non-fermentation-associated protein kinase
Y2H: yeast two-hybrid
Y1H: yeast one-hybrid
MYST, MOZ, YbF2: Sas2 and Tip60-like
HAT: histone acetyltransferase
BiFC: bimolecular fluorescence complementation
co-IP: co-immunoprecipitation
pPKD: phospho(Ser/Thr) PKD substrate antibody
ChIP: chromatin immunoprecipitation
CIP: calf intestinal alkaline phosphatase
anti-H4ace: H4 hyperacetylation (K5K8K12K16) antibody.
