# Supplementary material for: Epigenetic Regulation of Plant Tolerance to Salt Stress by Histone Acetyltransferase GsMYST1 From Wild Soybean
Source: Front Plant Sci. 2022 May 25;13:860056. doi: 10.3389/fpls.2022.860056 (PMC9174996; doi:10.3389/fpls.2022.860056)
Supplement: Supplementary file 1 [file Data_Sheet_1.PDF]

Figure legends of supplementary data

Figure S1. Subcellular localization of GsMYST1.

(A) Prediction of GsMYST1 subcellular localizations. (B) Experimental examination of GsMYST1 subcellular localization. GFP-MYST1 is localized in nucleus.

Figure S2. Comparison of promoter sequences of *GsMYST1* and *GmMYST1* genes

The approximate 2 kb upstream sequences of GsMYST1 and GmMYST1 genes were extracted and compared by using ClustalW software.

Figure S3. Response of *GmMYST1* gene to abiotic stresses in cultivated soybean.

The 6-week-old cultivated soybean seedlings were treated with salt and dehydration stresses for the indicated time periods and the total RNA samples were extracted from the treated soybean roots for qRT-PCR analyses.

Figure S4. Response of *GsMYST1* gene to salt stress.

GsMYST1pro::GUS was transformed into Arabidopsis. The transgenic plants were applied to 50 mM NaCl prior to GUS staining and (B) quantitative measurement of GUS activities.

Figure S5. Interaction of GsSnRK1 and GsMYST1

BD-*GsSnRK1* and AD-*GsMYST1* genes were co-transformed into yeast cells for yeast two-hybrid analyses.

Figure S6. Screening of GsMYST1 interactors

GsMYST1 was used as bait to screen wild soybean cDNA library. The representative positive yeast clones were shown. (B) The obtained putative interactors of GsMYST1.

Figure S7. Basic features of GsNAC83 protein

(A) Schematic representation of GsNAC83 protein. (B) Prediction of subcellular localization of GsNAC83. (C) Experimental examination of GsNAC83 subcellular localization. GFP-GsNAC83 is localized in nucleus.

Figure S8. Determination of GsSnRK1-GsMYST1-GsNAC83 ternary complex.

Myc-GsSnRK1, HA-GsMYST1 and Flag-GsNAC83 were co-transformed in plant cells and the co-immunoprecipitation assays were performed using the indicated antibodies.

Figure S9. Expression of GsSnRK1 and GsMYST1 in soybean hairy roots.

GsSnRK1, GsMYST1 and their mutants were co-transformed into soybean hairy roots. Total proteins were extracted for WB by using the indicated antibodies to determine the protein expression.

Figure S10. Overexpression of *GFP-GsMYST1* and silencing of *GmNAC83* in soybean hairy roots.

GFP-GsMYST1 overexpressing and *GmMYST1* RNAi-silencing constructs were transformed into soybean hairy roots. Total RNA samples were extracted for qRT-PCR analyses. Bars indicate SE, *t* test, and the columns were shown from three biological triplicates.

Figure S11. Examination of GFP-GsMYST1 expression in soybean.

Total proteins were extracted from soybean hairy roots and were applied for WB using anti-GFP antibody.

Figure S12. Effect of acetylation of *COR15B* gene by GsMYST1

Total proteins were extracted from soybean hairy roots transformed with GsMYST1 overexpressing and RNAi-silencing constructs for WB by using anti-H4ace and anti-H4 antibodies. (B) Quantitative measurement of acetylation from above. Bars indicate SE, *t* test, and the columns were shown from three biological triplicates.

Figure S13. Comparison of GsNAC83 and AtVIN2 sequences

The sequences of GsNAC83 and AtVIN2 proteins were aligned using NCBI sequence alignment tool.

Figure S14. Expression of GFP-GsNAC83 in soybean hairy roots

Total proteins were extracted from soybean hairy roots and were applied for WB using anti-GFP antibody.

Figure S15. Prediction of GsNAC83 binding sites on *COR15B* gene

Figure S16. Examination of GsNAC83 binding to ACATG box by yeast one-hybrid assay

Figure S17. Constructs for examination of *COR15B* expression regulated by GsSnRK1-GsMYST1-GsNAC83 module

Figure S18. Comparison of promoter sequences of *GsCOR15B* and *GmCOR15B* genes

Table S1. List of primers for qRT-PCR and PCR analyses

Table S2. The predicted *cis*-acting elements on the promoter of *GsMYST1* gene

Table S3. Prediction of phosphorylation sites on GsMYST1 protein by GsSnRK1.

Table S4. Prediction of phosphorylation sites on GsNAC83 protein by GsSnRK1
