# Supplementary material for: Epigenetic Regulation of Plant Tolerance to Salt Stress by Histone Acetyltransferase GsMYST1 From Wild Soybean
Source: Front Plant Sci. 2022 May 25;13:860056. doi: 10.3389/fpls.2022.860056 (PMC9174996; doi:10.3389/fpls.2022.860056)
Supplement: Supplementary file 2 [file Data_Sheet_2.PDF]

Figure S1

A

```
5712 multiple located sequences are accepted
ProtComp Version 9.0. Identifying sub-cellular location (Animals&Fungi)
Seq name:  test sequence, Length=435
Significant similarity in Location DB - Nuclear
Database sequence: AC=O94446 Location:Nuclear  DE  Histone acetyltransferase mst1;
Score=67, Sequence length=464, Alignment length=295
Predicted by Neural Nets - Cytoplasmic with score    2.7
Integral Prediction of protein location: Nuclear with score    9.9
Location weights:     LocDB / PotLocDB / Neural Nets / Pentamers / Integral
 Nuclear               10.0 /      3.0 /        0.74 /      0.25 /     9.92
 Plasma membrane        0.0 /      0.0 /        0.05 /      0.00 /     0.00
 Extracellular          0.0 /      0.0 /        0.00 /      0.25 /     0.00
 Cytoplasmic            0.0 /      0.0 /        2.71 /      0.07 /     0.00
 Mitochondrial          0.0 /      0.0 /        0.11 /      0.60 /     0.00
 Endoplasm. retic.      0.0 /      0.0 /        0.00 /      0.00 /     0.00
 Peroxisomal            0.0 /      0.0 /        0.00 /      0.00 /     0.00
 Lysosomal              0.0 /      0.0 /        0.06 /      0.00 /     0.00
 Golgi                  0.0 /      0.0 /        0.03 /      2.02 /     0.08
 Vacuolar               0.0 /      0.0 /        0.00 /      0.02 /     0.00


The protein is possibly multilocated: Cytoplasm_and_Nucleus due to SBLAST search in MultiLocDB
******************************************
```

Figure S1

B

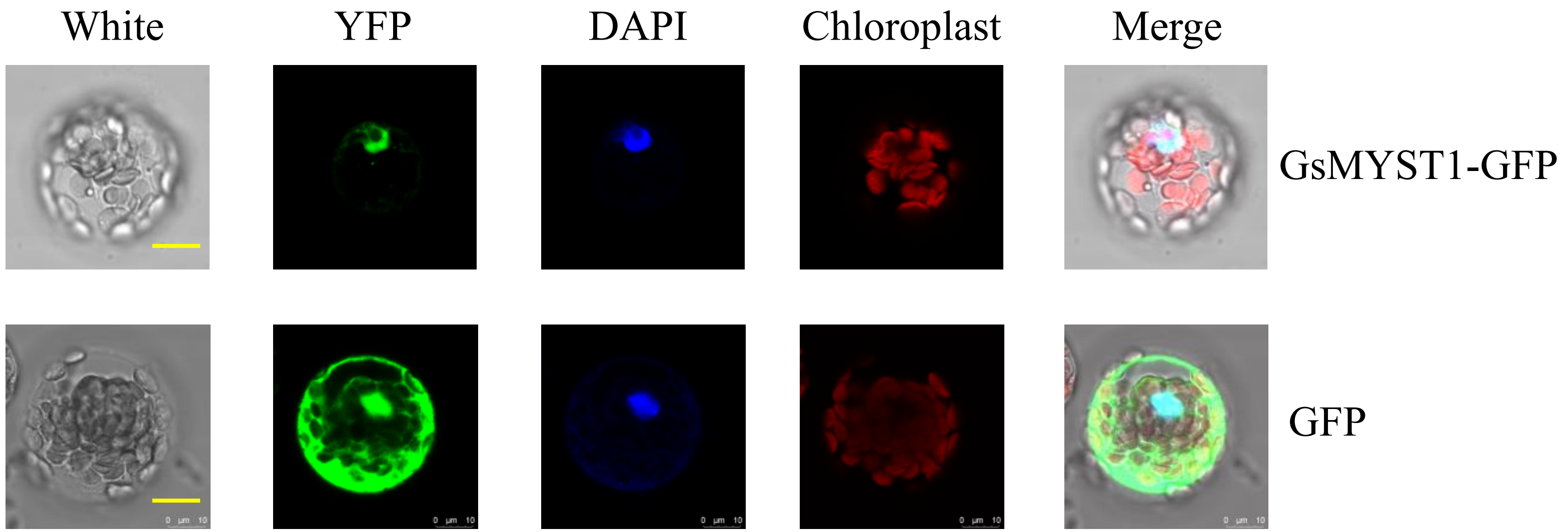

Figure S2

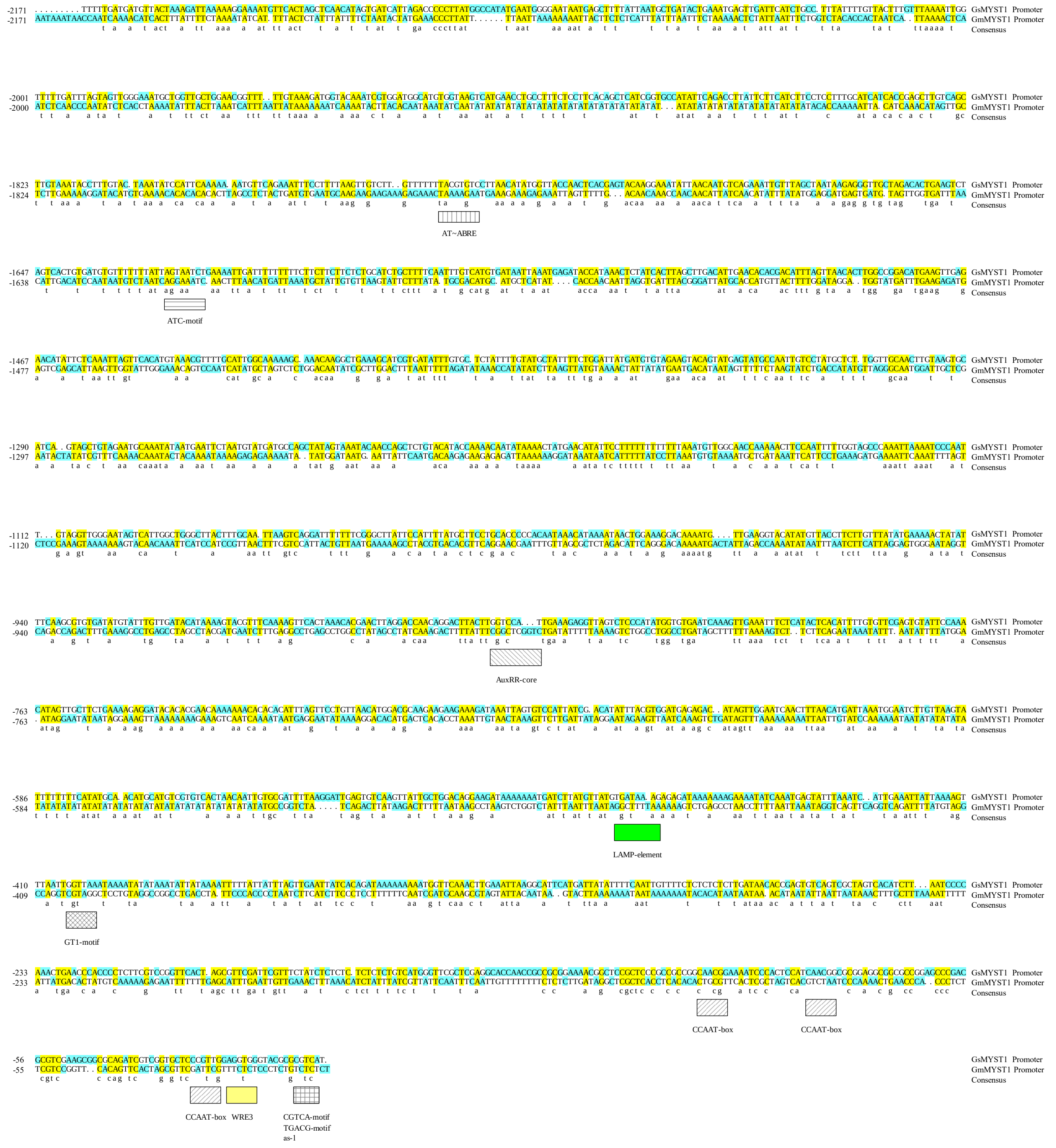
GsMYST1 Promoter
GmMYST1 Promoter
Consensus
AT~ABRE
ATC-motif
AuxRR-core
LAMP-element
GT1-motif
CCAAT-box
CCAAT-box
CCAAT-box
WRE3
CGTCA-motif
TGACG-motif
as-1

Figure S3

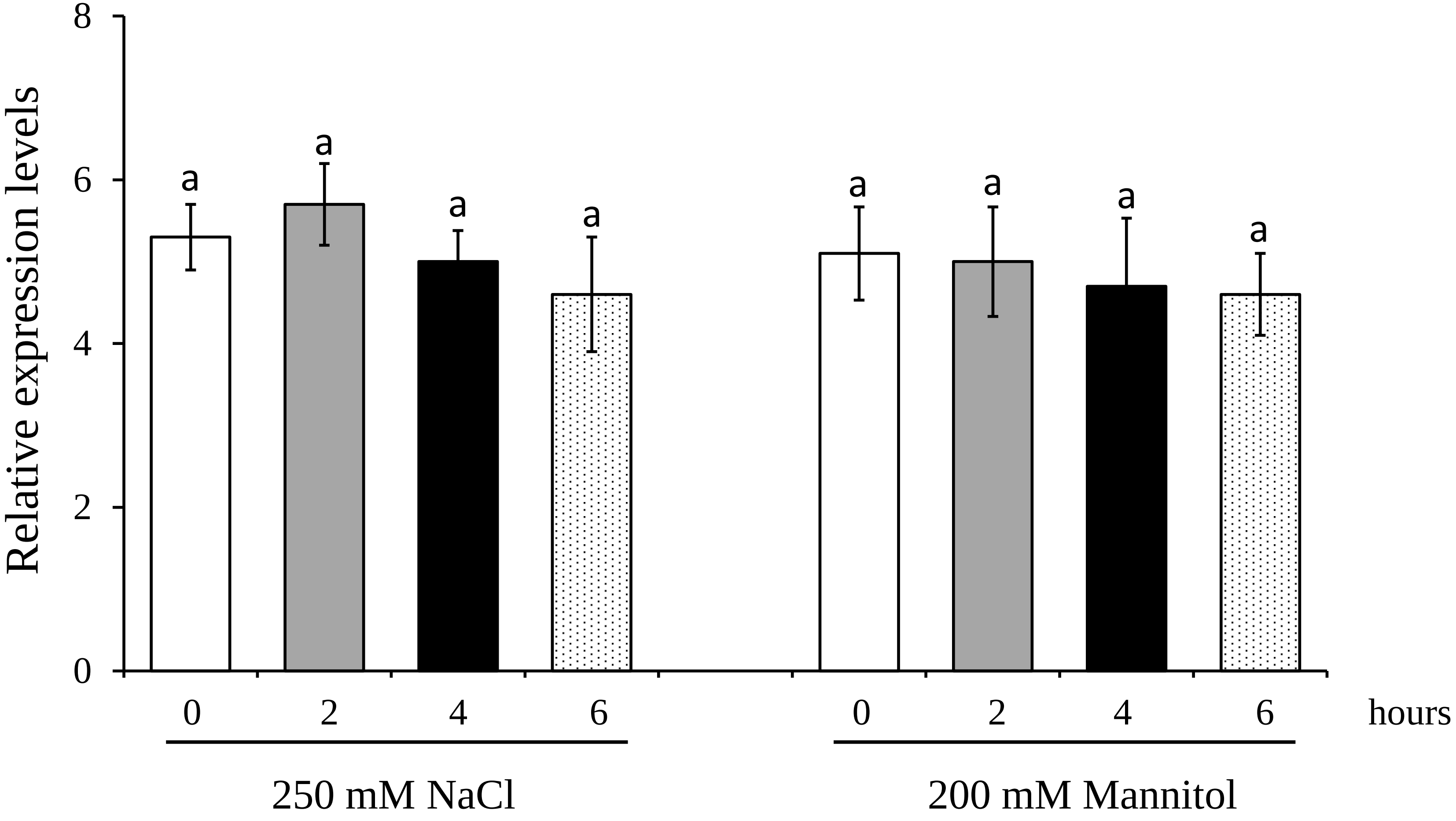

Relative expression levels
8
6
4
2
0
a
a
a
a
a
a
a
a
0
2
4
6
0
2
4
6
hours
250 mM NaCl
200 mM Mannitol

Figure S4

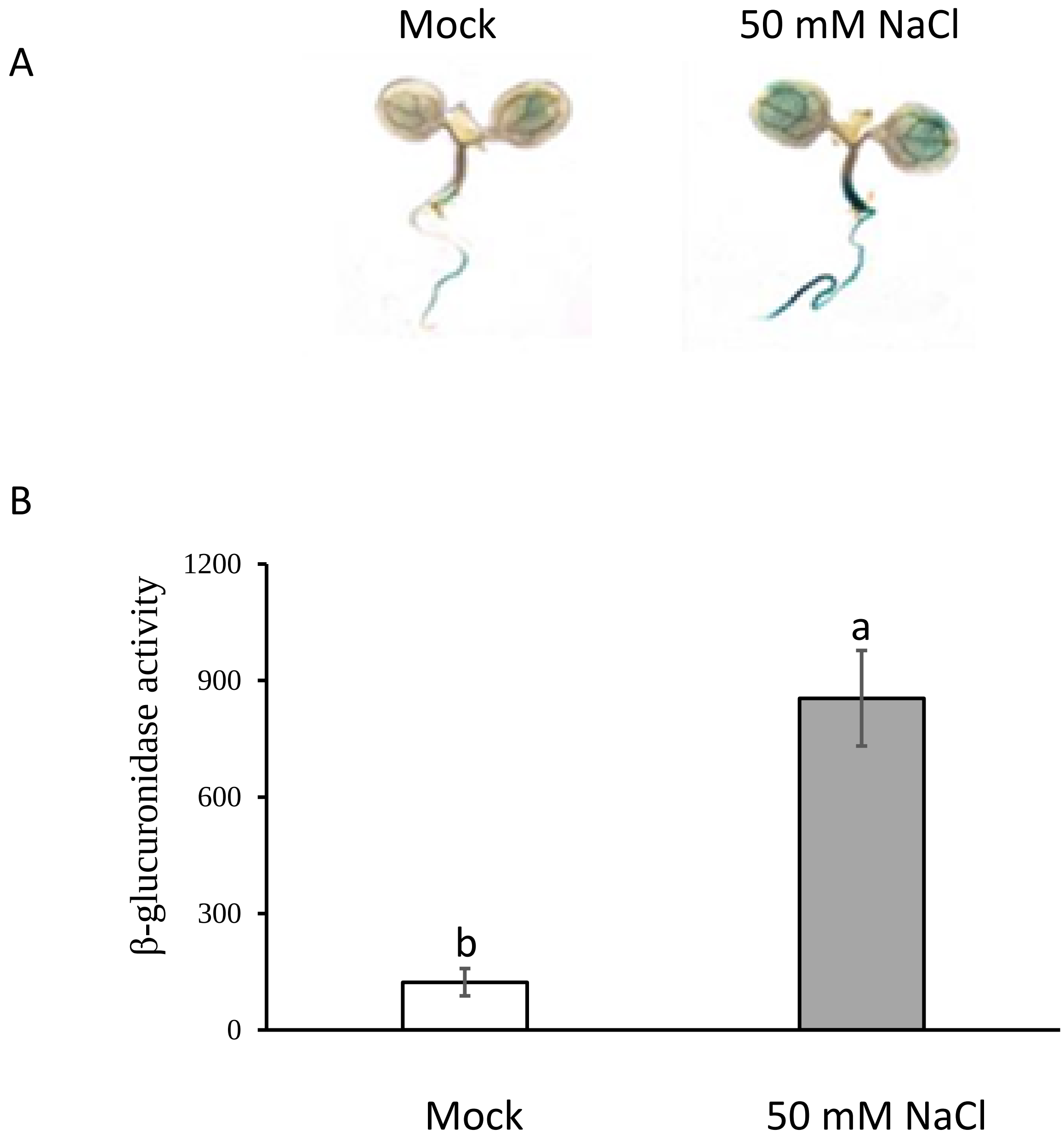
A
Mock
50 mM NaCl
B
β-glucuronidase activity
1200
900
600
300
0
a
b
Mock
50 mM NaCl

Figure S5

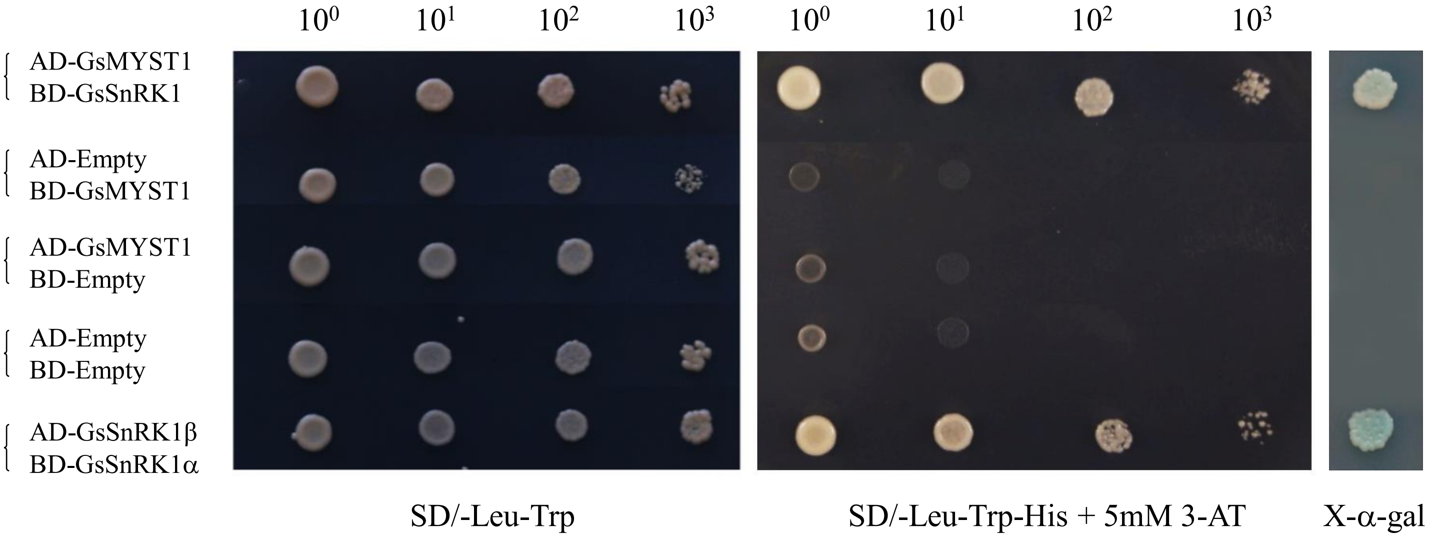

10⁰
10¹
10²
10³
10⁰
10¹
10²
10³
AD-GsMYST1
BD-GsSnRK1
AD-Empty
BD-GsMYST1
AD-GsMYST1
BD-Empty
AD-Empty
BD-Empty
AD-GsSnRK1β
BD-GsSnRK1α
SD/-Leu-Trp
SD/-Leu-Trp-His + 5mM 3-AT
X-α-gal

Figure S6

A

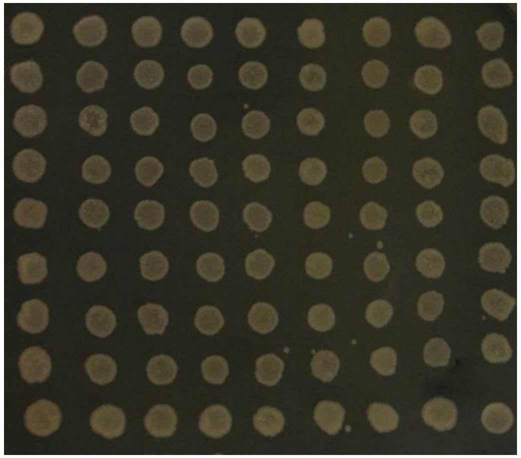

SD/-Leu-Trp

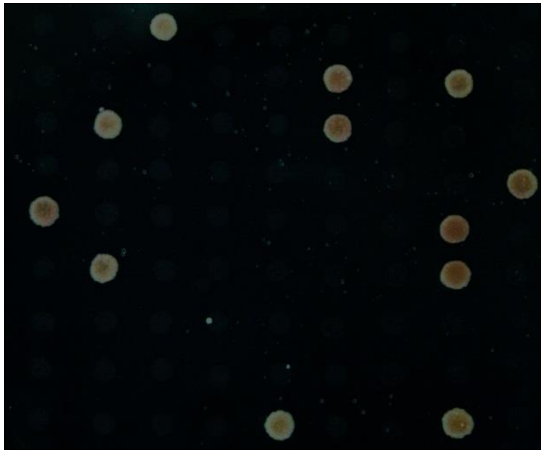

SD/-Leu-Trp-His + 5 mM 3-AT

B

Bait	Preys	ID
GsMYST1	GsNAC83	Glysoja.05G012860.1
	GsNHL13 NDR1/HIN1-like protein 13	Glysoja.14G037661.1
	Calcium-dependent lipid-binding family protein	Glysoja.14G039496.1
	TESMIN/TSO1-related protein	Glysoja.08G019413.2

Figure S7

A

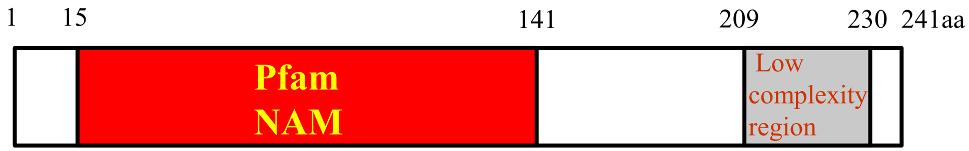

B

5724 multiple located sequences are accepted
ProtComp Version 9.0. Identifying sub-cellular location (Plant)
Seq name: test sequence, Length=241
Significant similarity in Location DB - Nuclear
Database sequence: AC=Q9C932 Location:Nuclear DE NAC domain-containing protein 1
Score=58, Sequence length=318, Alignment length=141
Predicted by Neural Nets - Extracellular (Secreted) with score 1.0
Integral Prediction of protein location: Nuclear with score 8.8

Location weights:	LocDB /	PotLocDB /	Neural Nets /	Pentamers /	Integral
Nuclear	10.0 /	3.0 /	0.00 /	0.00 /	8.81
Plasma membrane	0.0 /	0.0 /	0.96 /	0.15 /	0.73
Extracellular	0.0 /	0.0 /	0.96 /	2.06 /	0.01
Cytoplasmic	0.0 /	0.0 /	0.00 /	0.85 /	0.00
Mitochondrial	0.0 /	0.0 /	0.00 /	2.21 /	0.06
Endoplasm. retic.	0.0 /	0.0 /	0.00 /	0.18 /	0.00
Peroxisomal	0.0 /	0.0 /	0.96 /	0.00 /	0.07
Golgi	0.0 /	0.0 /	0.11 /	0.00 /	0.00
Chloroplast	0.0 /	0.0 /	0.00 /	0.25 /	0.05
Vacuolar	0.0 /	0.0 /	0.00 /	0.00 /	0.27

SoftBerry

Figure S7

C

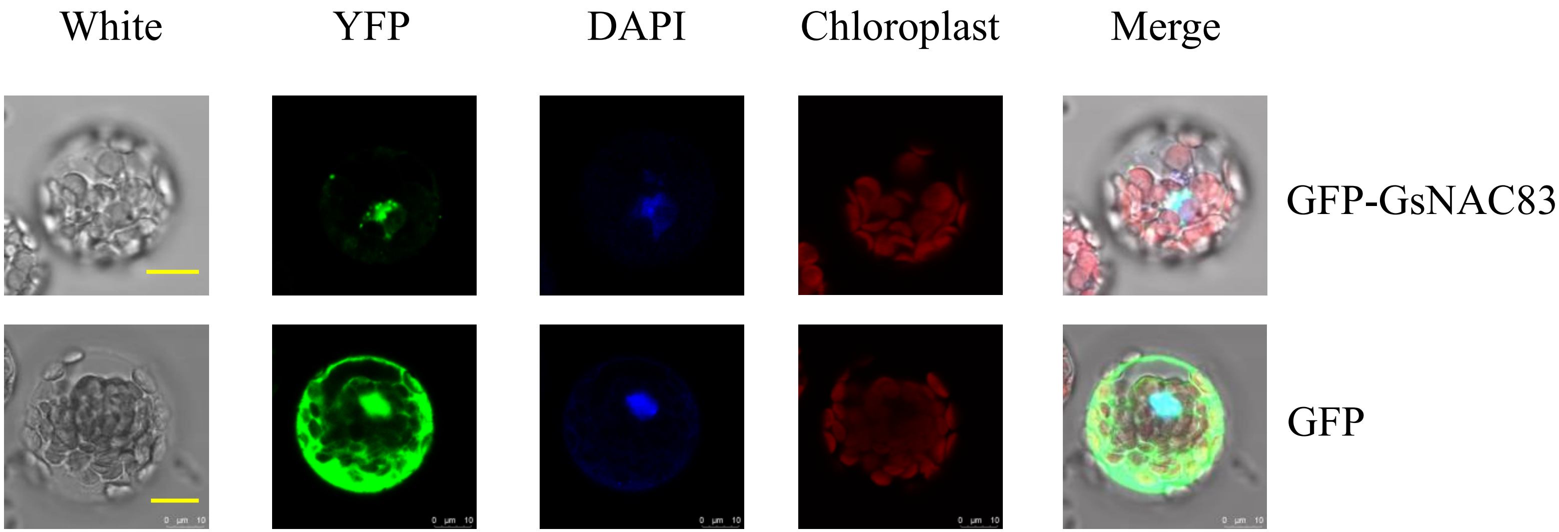

Figure S8

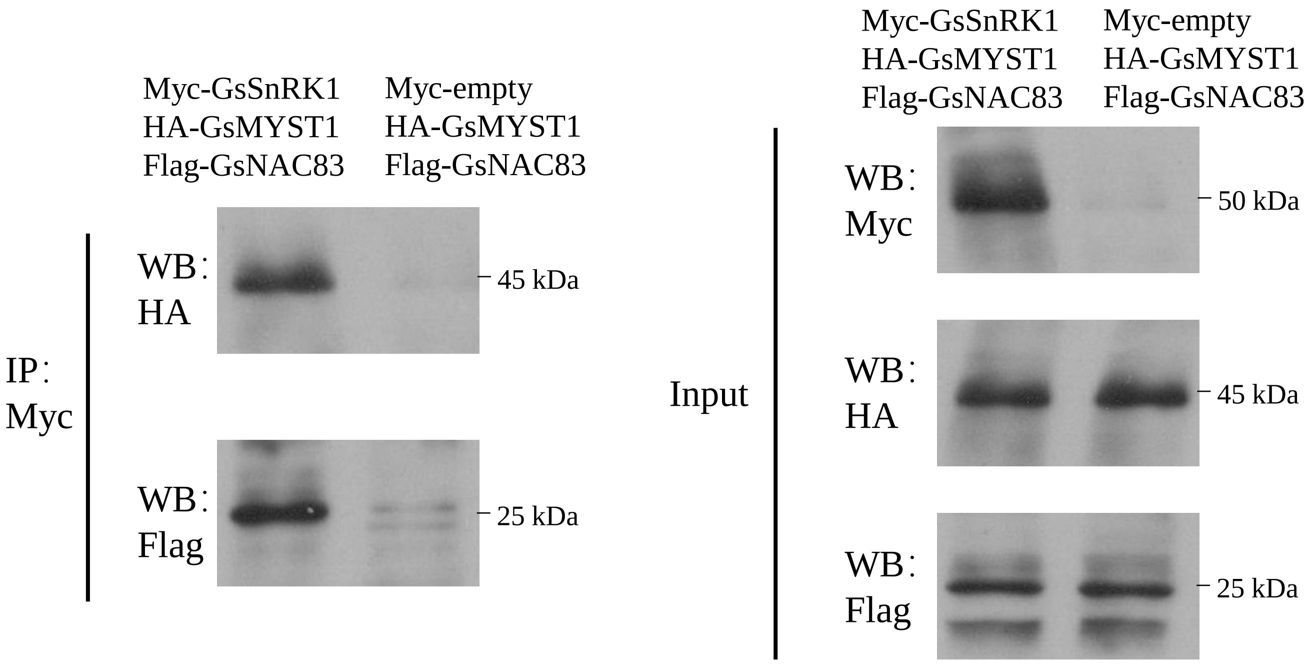
Myc-GsSnRK1
HA-GsMYST1
Flag-GsNAC83
Myc-empty
HA-GsMYST1
Flag-GsNAC83
IP：
Myc
WB：
HA
45 kDa
WB：
Flag
25 kDa
Input
WB：
Myc
50 kDa
WB：
HA
45 kDa
WB：
Flag
25 kDa

Figure S9

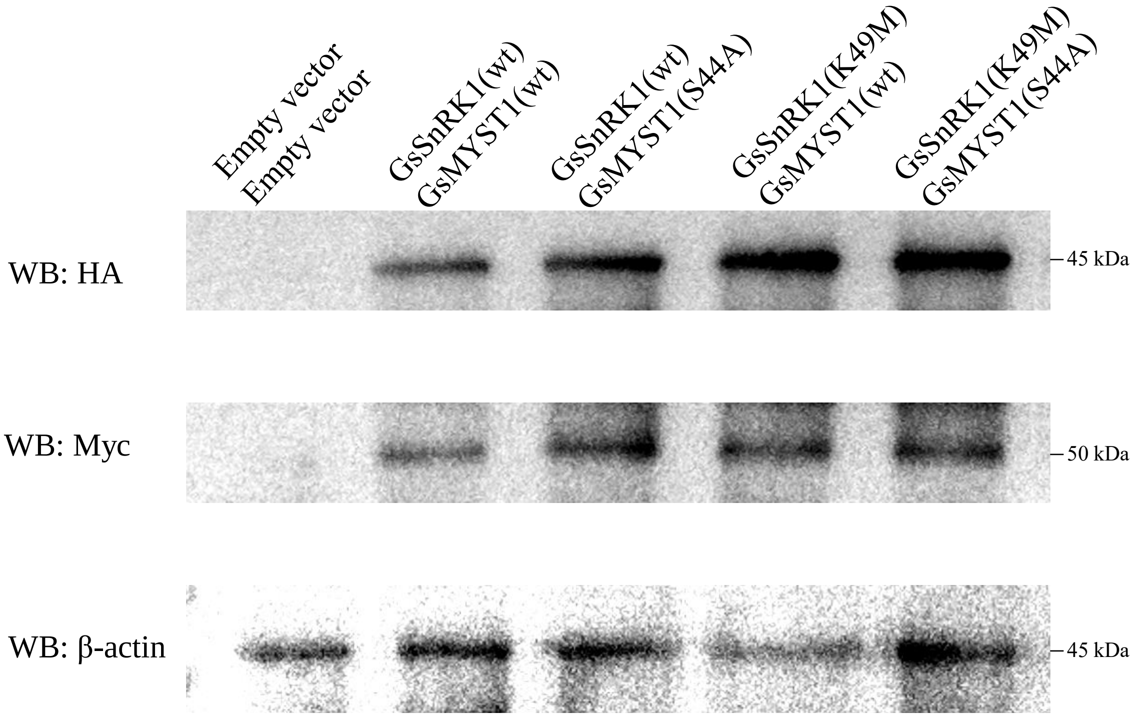
Empty vector
Empty vector
GsSnRK1(wt)
GsMYST1(wt)
GsSnRK1(wt)
GsMYST1(S44A)
GsSnRK1(K49M)
GsMYST1(wt)
GsSnRK1(K49M)
GsMYST1(S44A)
WB: HA
45 kDa
WB: Myc
50 kDa
WB: β-actin
45 kDa

Figure S10

A

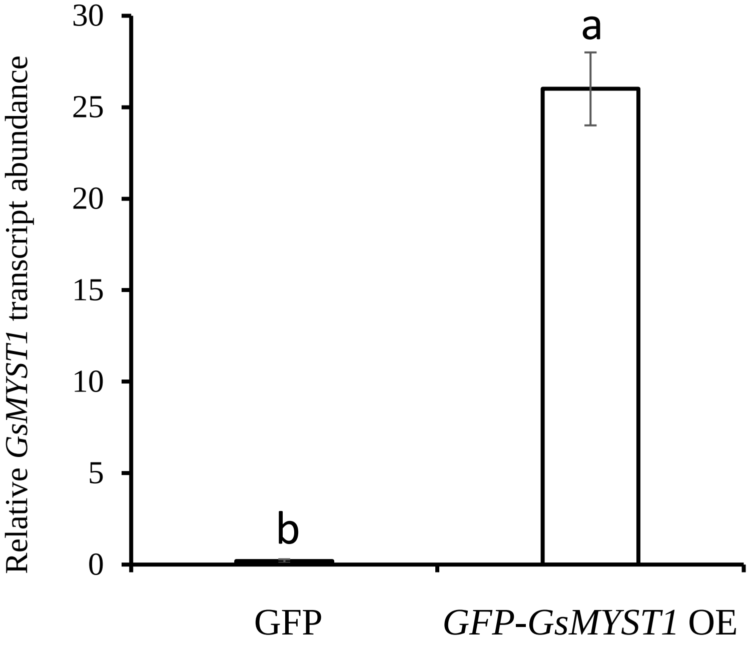

30
25
20
15
10
5
0
Relative GsMYST1 transcript abundance
a
b
GFP
GFP-GsMYST1 OE

Figure S10

B

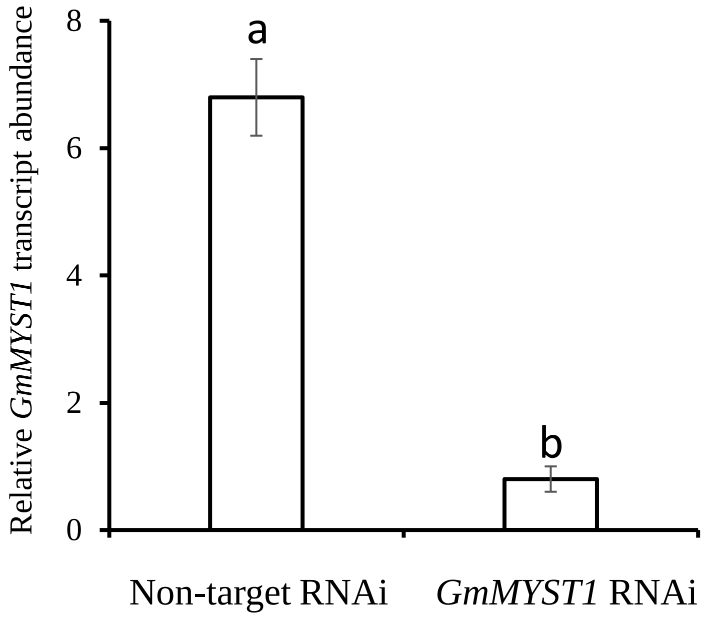
Relative GmMYST1 transcript abundance
8
6
4
2
0
a
b
Non-target RNAi
GmMYST1 RNAi

Figure S11

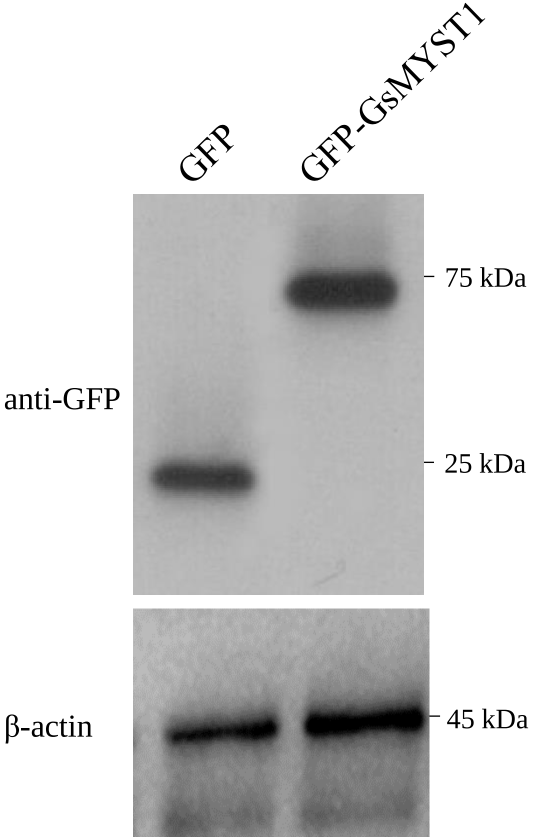

Figure S12

A

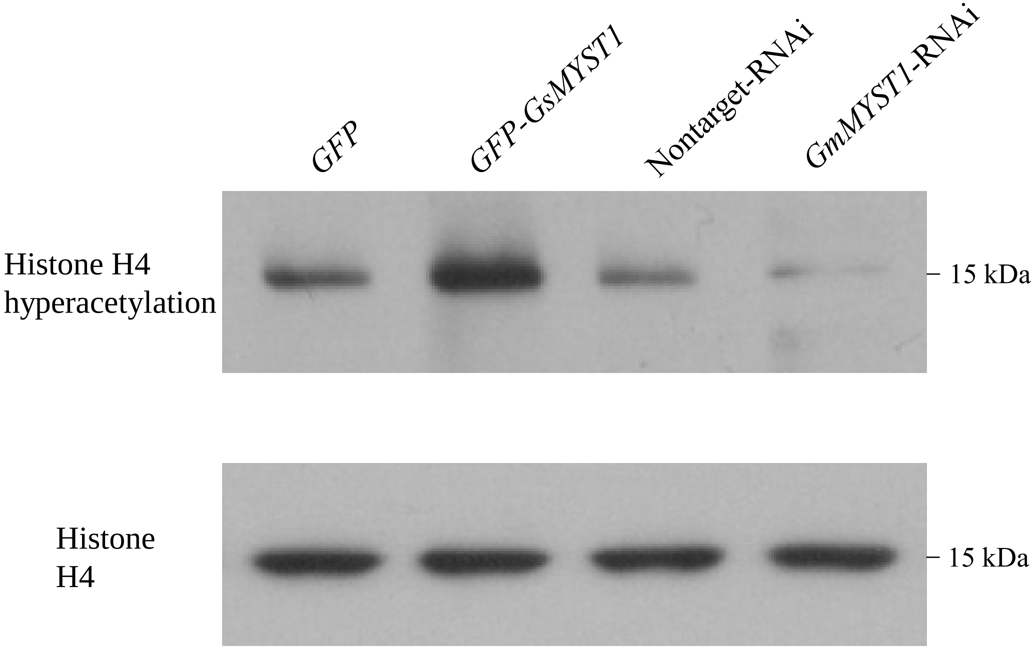

GFP
GFP-GsMYST1
Nontarget-RNAi
GmMYST1-RNAi
Histone H4 hyperacetylation
15 kDa
Histone H4
15 kDa

Figure S12

B

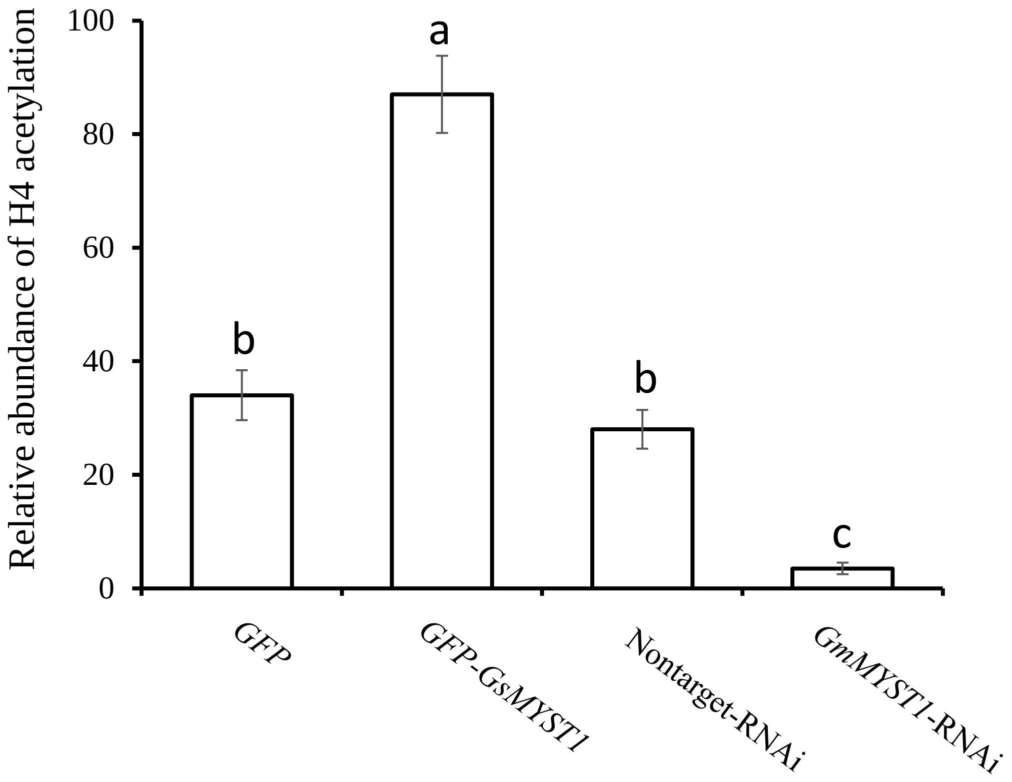

Relative abundance of H4 acetylation
100
80
60
40
20
0
a
b
b
c
GFP
GFP-GsMYST1
Nontarget-RNAi
GmMYST1-RNAi

Figure S13

```
GsNAC83    MEKLNFVKNGVSKLPPGFRFQPTDEELVFQYLKCKVFSYPLPASIIPEINVCKYDPWDLPGNCDPQERHFFSPKEAKYRNGNRMNRTTKCGYWKATGSDK   100
AtVNI2     MDNVKLVKNGVLRLPPGFRFHPTDEELVVQYLKRKVCSSPLPASIIPEFDVCRADPWDLPGNLE.KERYFFSTREAKYPNGNRSNRATGSGYWKATGIDK    99
Consensus  m     vkngv  lppgfrf ptdeelv qylk kv s plpasiipe  vc  dpwdlpgn    er ffs  eaky ngnr nr t  gywkatg dk

GsNAC83    RISSSTSTCNGIVGVRKTLIFYEGKSPKGSRTHWVLHEYRLVSVETGAANSSHNYVNEIGDWVLCRLSMKKRS....VESDGSGTHHKHRQN........   188
AtVNI2     RVVTSRG..NQIVGLKKTLVFYKGKPPHGSRTDWIMHEYRLSSSPPSSMGPTQN.......WVLCRIFLKKRAGNKNDDDDGDSRNLRHNNNNNSSDQIE   190
Consensus  r   s    n ivg  ktl fy gk p gsrt w  heyrl s          n       wvlcr   kkr        dg      h  n

GsNAC83    ....TAVQTTRPRLMFDFMMVGKTNSS....TSSSCSSSSNIMEVSSNASDHEETSGYAHF                                         241
AtVNI2     IITTDQTDDKTKPIFFDFMRKERTTDLNLLPSSPSSDHASSGVTTEIFSSSDEETSSCNSF                                         251
Consensus                 fdfm    t        s s    s         s  eets    f
```

Figure S14

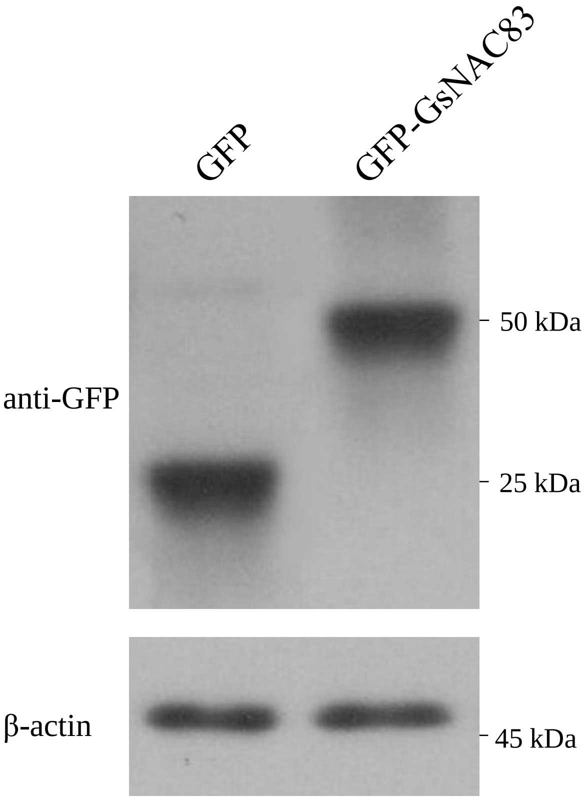

Figure S15

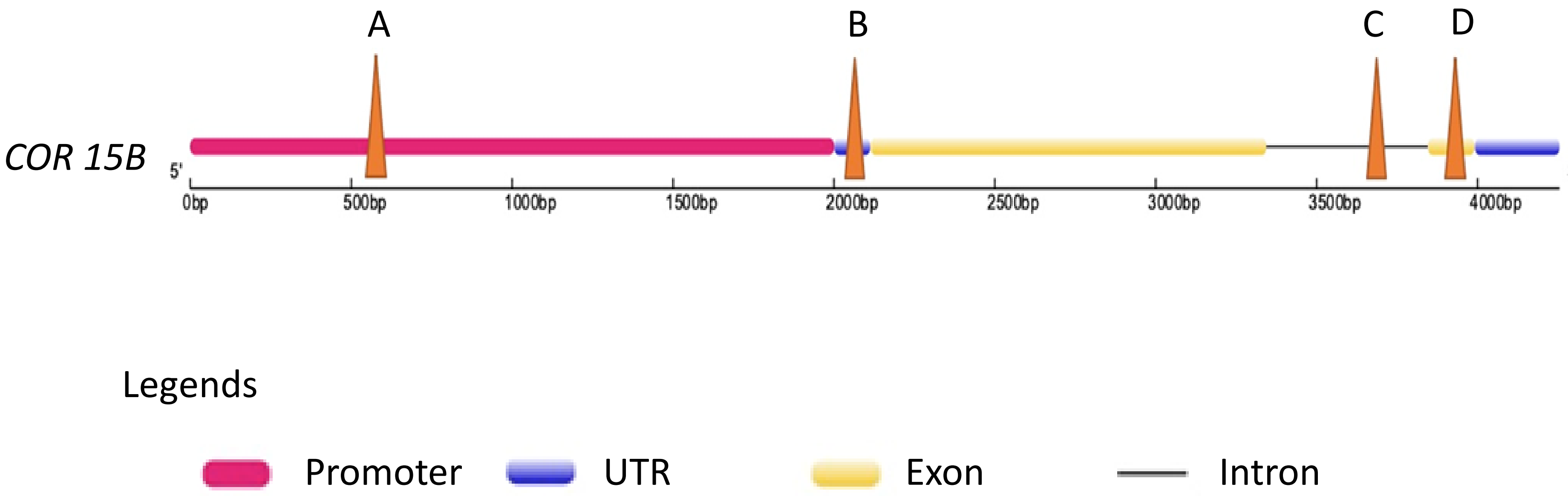
A
B
C
D
COR 15B
5'
3'
0bp
500bp
1000bp
1500bp
2000bp
2500bp
3000bp
3500bp
4000bp
Legends
Promoter
UTR
Exon
Intron

Figure S16

COR15B BS -1611 TAGAT**ACATG**TTGCA

COR15B mBS TAGAT**CCCCC**TTGCA

10^0 10^1 10^2 10^3 10^0 10^1 10^2 10^3

AD-GsNAC83
+pHIS2-BS

AD-GsNAC83
+pHIS2-mBS

AD-Empty
+pHIS2-BS

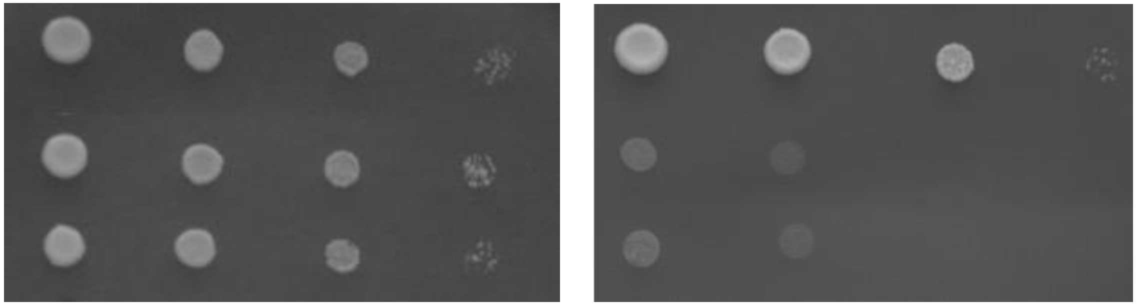

SD/-Leu-Trp SD/-Leu-Trp-His + 20 mM 3-AT

Figure S17

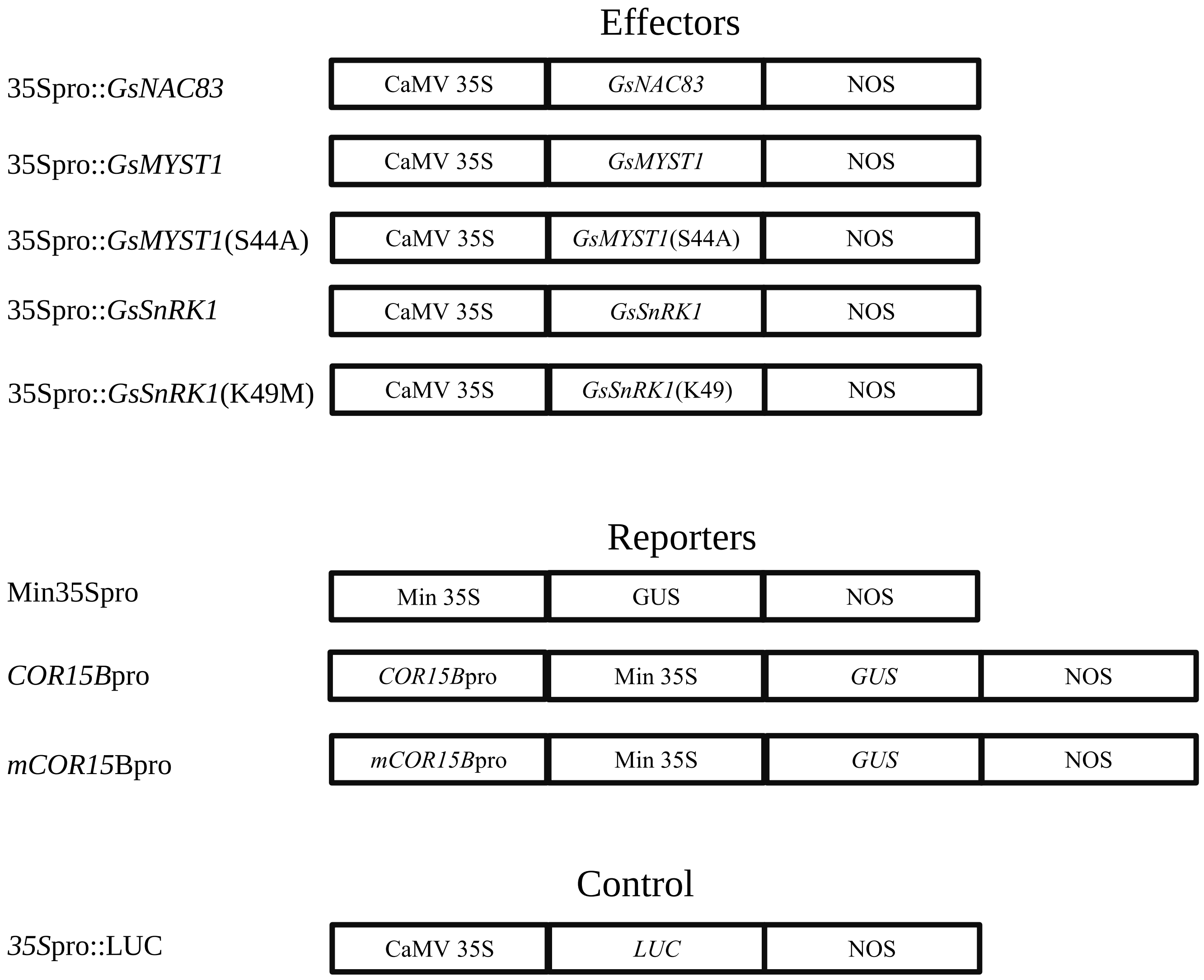
Effectors
35Spro::*GsNAC83*
CaMV 35S | *GsNAC83* | NOS
35Spro::*GsMYST1*
CaMV 35S | *GsMYST1* | NOS
35Spro::*GsMYST1*(S44A)
CaMV 35S | *GsMYST1*(S44A) | NOS
35Spro::*GsSnRK1*
CaMV 35S | *GsSnRK1* | NOS
35Spro::*GsSnRK1*(K49M)
CaMV 35S | *GsSnRK1*(K49) | NOS
Reporters
Min35Spro
Min 35S | GUS | NOS
*COR15B*pro
*COR15B*pro | Min 35S | *GUS* | NOS
*mCOR15B*pro
*mCOR15B*pro | Min 35S | *GUS* | NOS
Control
*35S*pro::LUC
CaMV 35S | *LUC* | NOS

Figure S18

Position	Sequence	
-2114	TGGAATTATATGGTTATTTTTACCAGTAACTGATACAAGAAATCTGAAACATTTGTTTGCACAGGGAATCGTTTTTTAATCAAAATGCACATCTCTGAGCTATTTCCAACAACAGTACAGAATTGAGATATATTTTAGTGGCTGCATAGGATTTGATTCAGATCTCCAAAATTCTTGTGC	GsCOR15B Promoter
-2113	TGGAATTATATGGTTATTTTTACCAGTAACTGATACAAGAAATCTGAAACATTTGTTTGCACAGGGAATCGTTTTTTAATCAAAATGCACATCTCTGAGCTATTTCCAACAACAGTACAGAATTGAGATATATTTTAGTGGCTGCATAGGATTTGATTCAGATCTCCAAAATTCTTGTGC	GmCOR15B Promoter
	tggaattatatggttatttttaccagtaactgatacaagaaatctgaaacatttgtttgcacagggaatcgttttttaatcaaaatgcacatctctgagctatttccaacaacagtacagaattgagatatattttagtggctgcataggatttgattcagatctccaaaattcttgtgc	Consensus
-1934	TGATGATCTAGAGATTATCAAATTGCGATATTAGTTAGTTAGTTAGTCAGTTTTTCTCCTTTTGCCCTATCCAATGTTATTTGCCAGGGAACAAATTACTTGGTCTTGAGAAGTGTATATATTTTGTTAAGATAGAACAGAAACCAAGAGTTCTCAGAGTAGGCTTTCCTTGTTACGATT	
-1933	TGATGATCTAGAGATTATCAAATTGCGATATTAGTTAGTTAGTTAGTCAGTTTTTCTCCTTTTGCCCTATCCAATGTTATTTGCCAGGGAACAAATTACTTGGTCTTGAGAAGTGTATATATTTTGTTAAGATAGAACAGAAACCAAGAGTTCTCAGAGTAGGCTTTCCTTGTTACGATT	
	tgatgatctagagattatcaaattgcgatattagttagttagttagtcagtttttctccttttgccctatccaatgttatttgccagggaacaaattacttggtcttgagaagtgtatatattttgttaagatagaacagaaaccaagagttctcagagtaggctttccttgttacgatt	
-1754	TTCTATTTATTTAATGGAATCTAGTTCTTGGCTAAAGAACTAGATGCCTCCTTAACTTGGATGTTACCTTGGCTTGTGATTTTCTTATGGAGCCATTATAGGAGATGCCTCCTTAACTTCATCATGACCGGTGGAAGACATCTAGATACATGTTGCATACAATACAAGCTGTAAGAGTGC	GsCOR15B Promoter
-1753	TTCTATTTATTTAATGGAATCTAGTTCTTGGCTAAAGAACTAGATGCCTCCTTAACTTGGATGTTACCTTGGCTTGTGATTTTCTTATGGAGCCATTATAGGAGATGCCTCCTTAACTTCATCATGACCGGTGGAAGACATCTAGATACATGTTGCATACAATACAAGCTGTAAGAGTGC	GmCOR15B Promoter
	ttctatttatttaatggaatctagttcttggctaaagaactagatgcctccttaacttggatgttaccttggcttgtgattttcttatggagccattataggagatgcctccttaacttcatcatgaccggtggaagacatctagatacatgttgcatacaatacaagctgtaagagtgc	Consensus
-1574	TTATGTGAAGTGTCTTTTGCATACCTCACTGAATGCAAAATTTATAATCATTATAGCTCTTTCTTAATGATATTTAAAGGTATTTTCATATGATTTCCTTTCTCTGTTTCGCGAGGCAAATTTATCAAACAGGTGTAGATTTGATTTCAGCCCAAATAGAGCAAACGATCAATTTAGTCT	
-1573	TTATGTGAAGTGTCTTTTGCATACCTCACTGAATGCAAAATTTATAATCATTATAGCTCTTTCTTAATGATATTTAAAGGTATTTTCATATGATTTCCTTTCTCTGTTTCGCGAGGCAAATTTATCAAACAGGTGTAGATTTGATTTCAGCCCAAATAGAGCAAACGATCAATTTAGTCT	
	ttatgtgaagtgtcttttgcatacctcactgaatgcaaaatttataatcattatagctctttcttaatgatatttaaaggtattttcatatgatttcctttctctgtttcgcgaggcaaatttatcaaacaggtgtagatttgatttcagcccaaatagagcaaacgatcaatttagtct	
-1394	CTAATTTCATATCTTACTGTTTAAAAGTAAGGAAATTCAAGTAATTTCCTGAATTTTTTTAGGATTCCATTTAAGTTCTGGCATTAATCTCTTTTTTTTTTTTTTCATTCTGGTTCCTAGTTTGGGAACTCAAGTTAAGGGACCAAAGAGAAAAAAAGATTTAACTTCAGGGACTTAAATG	GsCOR15B Promoter
-1393	CTAATTTCATATCTTACTGTTTAAAAGTAAGGAAATTCAAGTAATTTCCTGAATTTTTTTAGGATTCCATTTAAGTTCTGGCATTAATCTCTTTTTTTTTTTTT.CATTCTGGTTCCTAGTTTGGGAACTCAAGTTAAGGGACCAAAGAGAAAAAAAGATTTAACTTCAGGGACTTAAATG	GmCOR15B Promoter
	ctaatttcatatcttactgtttaaaagtaaggaaattcaagtaatttcctgaatttttttaggattccatttaagttctggcattaatctcttttttttttttt cattctggttcctagtttgggaactcaagttaagggaccaaagagaaaaaaagatttaacttcagggacttaaatg	Consensus
-1214	AAATCCTACAAAATTTTGGGACTCATTTGTGTTTTCTTTAGTTCTTGAGACTAAAATGGTAGCAAGATAAAATTCATGGATTAAATTTATCATTTTTGTAAATTAAATTATTTATCATTTTTTTCTCATTATTTTATAATTTAAGAAACTAAAAATAAAAGGAGAGATCTCTTGACATG	
-1214	AAATCCTACAAAATTTTGGGACTCATTTGTGTTTTCTTTAGTTCTTGAGACTAAAATGGTAGCAAGATAAAATTCATGGATTAAATTTATCATTTTTGTAAATTAAATTATTTATCATTTTTTTCTCATTATTTTATAATTTAAGAAACTAAAAATAAAAGGAGAGATCTCTTGACATG	
	aaatcctacaaaattttgggactcatttgtgttttctttagttcttgagactaaaatggtagcaagataaaattcatggattaaatttatcatttttgtaaattaaattatttatcatttttttctcattattttataatttaagaaactaaaaataaaaggagagatctcttgacatg	
-1034	TCAATTTGGGAATTTCTCTTTTTCAAACAATGAAAAATTGAATTTTTTTTAATATTTATTTGCTTTTGACTTTCTTTTCCAGTGAAAAATAGTGTTCCACCTTGGCGATATTCTGATTATTATTTATGTCGTAGTTTCAGTTTGATTCGTGATGTAAGCTGACTGGGTCGTGCCATGATT	GsCOR15B Promoter
-1034	TCAATTTGGGAATTTCTCTTTTTCAAACAATGAAAAATTGAATTTTTTTTAATATTTATTTGCTTTTGACTTTCTTTTCCAGTGAAAAATAGTGTTCCACCTTGGCGATATTCTGATTATTATTTATGTCGTAGTTTCAGTTTGATTCGTGATGTAAGCTGACTGGGTCGTGCCATGATT	GmCOR15B Promoter
	tcaatttgggaatttctctttttcaaacaatgaaaaattgaattttttttaatatttatttgcttttgactttcttttccagtgaaaaatagtgttccaccttggcgatattctgattattatttatgtcgtagtttcagtttgattcgtgatgtaagctgactgggtcgtgccatgatt	Consensus
-854	CAATAAGCAACTTGTAAACAATTGACATATTTTTAATCCATTTTTCATTTACAAAAGAAATTAGGAAACATTAAGTAGTATATTACTAGTTTGAAGAACACATTTTATAACACACTCAATCTAGTACATACTCATTATTAAAAATCATTAATAAAGTTTATTAAATGTGGAGTAAAGTTT	
-854	CAATAAGCAACTTGTAAACAATTGACATATTTTTAATCCATTTTTCATTTACAAAAGAAATTAGGAAACATTAAGTAGTATATTACTAGTTTGAAGAACACATTTTATAACACACTCAATCTAGTACATACTCATTATTAAAAATCATTAATAAAGTTTATTAAATGTGGAGTAAAGTTT	
	caataagcaacttgtaaacaattgacatatttttaatccatttttcatttacaaaagaaattaggaaacattaagtagtatattactagtttgaagaacacattttataacacactcaatctagtacatactcattattaaaaatcattaataaagtttattaaatgtggagtaaagttt	
-674	ACATAAAAATTATTTATAATTTCTAATACACTTGAATAGTAATAAAAGTGTATTAAAAAAAAAGGTGTTTTTAATACAAATTTGAAACGGAAAATAAGCTGGTTGTCATGCATTTCTATAATTTATCATTAATGGGAGAAGCAAGAAAGCAATGTTAACTCGATAAGACAGAACATTGGAG	GsCOR15B Promoter
-674	ACATAAAAATTATTTATAATTTCTAATACACTTGAATAGTAATAAAAGTGTATTAAAAAAAAAGGTGTTTTTAATACAAATTTGAAACGGAAAATAAGCTGGTTGTCATGCATTTCTATAATTTATCATTAATGGGAGAAGCAAGAAAGCAATGTTAACTCGATAAGACAGAACATTGGAG	GmCOR15B Promoter
	acataaaaattatttataatttctaatacacttgaatagtaataaaagtgtattaaaaaaaaaggtgtttttaatacaaatttgaaacggaaaataagctggttgtcatgcatttctataatttatcattaatgggagaagcaagaaagcaatgttaactcgataagacagaacattggag	Consensus
-494	GCTTTTTGCATTCTCAAATGGAATTCTAGTTCTAGTTCTTTGGCGGTGGCAAGGAAAAAAATAAAAAGAAGCAAAAGCAATATAGAGATTAGAGAGTACAAGAAGCAGCCAGAAAAGAACTAGTTATCCATCCAATTCCAATCTACAATCCTTTTACTCGGTGCATCCCTGTTCCATCCG	
-494	GCTTTTTGCATTCTCAAATGGAATTCTAGTTCTAGTTCTTTGGCGGTGGCAAGGAAAAAAATAAAAAGAAGCAAAAGCAATATAGAGATTAGAGAGTACAAGAAGCAGCCAGAAAAGAACTAGTTATCCATCCAATTCCAATCTACAATCCTTTTACTCGGTGCATCCCTGTTCCATCCG	
	gctttttgcattctcaaatggaattctagttctagttctttggcggtggcaaggaaaaaaataaaaagaagcaaaagcaatatagagattagagagtacaagaagcagccagaaaagaactagttatccatccaattccaatctacaatccttttactcggtgcatccctgttccatccg	
-314	GGAATTGACACGTGCAAACTCGGAAAACTCAGCATAAGAACCAAATGATGACATGCAGTTTGATACCTTTCTTTCTTCAACTGACACGTAAAATTTGACATATTTTGTAACTAAGCCACGTTGCATGCATCACACCCCCCATGTTCATCAACTCACTCCACTCCCTTATAAAAACCACCC	GsCOR15B Promoter
-314	GGAATTGACACGTGCAAACTCGGAAAACTCAGCATAAGAACCAAATGATGACATGCAGTTTGATACCTTTCTTTCTTCAACTGACACGTAAAATTTGACATATTTTGTAACTAAGCCACGTTGCATGCATCACACCCCCCATGTTCATCAACTCACTCCACTCCCTTATAAAAACCACCC	GmCOR15B Promoter
	ggaattgacacgtgcaaactcggaaaactcagcataagaaccaaatgatgacatgcagtttgatacctttctttcttcaactgacacgtaaaatttgacatattttgtaactaagccacgttgcatgcatcacaccccccatgttcatcaactcactccactcccttataaaaaccaccc	Consensus
-134	CAATCAAAATCAACCTCCACCACCACACTTGCATTGTATTTCGTTTGTTGTTCAATCTAACAAACTTCTCTATATCGTTTACTTTTTCAACACACCTTACACACTTCTTTATTTGAGCCTTTCATATAAATAT	
-134	CAATCAAAATCAACCTCCACCACCACACTTGCATTGTATTTCGTTTGTTGTTCAATCTAACAAACTTCTCTATATCGTTTACTTTTTCAACACACCTTACACACTTCTTTATTTGAGCCTTTCATATAAATAT	
	caatcaaaatcaacctccaccaccacacttgcattgtatttcgtttgttgttcaatctaacaaacttctctatatcgtttactttttcaacacaccttacacacttctttatttgagcctttcatataaatat	
