# Supplementary material for: Epigenetic Regulation of Plant Tolerance to Salt Stress by Histone Acetyltransferase GsMYST1 From Wild Soybean
Source: Front Plant Sci. 2022 May 25;13:860056. doi: 10.3389/fpls.2022.860056 (PMC9174996; doi:10.3389/fpls.2022.860056)
Supplement: Supplementary file 3 [file Data_Sheet_3.PDF]

Supplementary Table S1. Sequences of oligos for PCR or RT-qPCR

Primer	Sequence（5’- 3’）	Accession number
GsMYST1-*Sma* IF	CCCGGGTGGTTCACTCGAGGCCCCAA	Glysoja.06G016367.1
GsMYST1-*Sma* IR	CCCGGGACTTTGTTCTTTATAAGGAGTCCAGATCAG	
GsMYST1-*Sal* IR	GTCGACTCAACTTTGTTCTTTATAAGGAGTCCAG	
AD-GsMYST1-*Sma* IF	GGCCAGTGAATTCCACCCGGGTTCACTCGAGGCCC	
AD-GsMYST1-*Sma* IR	CCGTATCGATGCCCACCCACTTTGTTCTTTATAAGGAGTCCAGATCAG	
GsMYST1pro-F	AATAAATAACCAATCAAAACATCACTT	
GsMYST1pro-R	TAGAGAGACAGAGGGAGAGAAA	
HA-GsMYST-F	CCCATACGACGTACCAGATTACGCTCACGGGGGTTCACTCGAGGC	
pET32b-HA-GsMYST1-*Eco* RVF	CGACAAGGCCATGGCGATGGAGTACCCATACGACGTAC	
pET32b-HA-GsMYST1-*Eco* RVR	TAGACCTGAGGAATATTTCTTGTTTCAAGCCTAGGCTTAAGCTC	
GsMYST1(S44A)-R1	CGCCGATCTCCGCCGCTTCGATGC	
GsMYST1(S44A)-F1	CGGCGGAGATCGGCGGTGCTCCCACTG	
3301GFP-*Nco* IF	ACACGGGGGACTCTTGACCATGGGTAGTAAAGGAGAAGAACTTTTCACTGGAGT	
3301GFP-*Pml* IR	GGTCACCTGTAATTCACACGTGTCACACGTGGTGGTGGTGGT	
3301-35S-HA-GsMYST1-*Nco* IF	ACACGGGGGACTCTTGACCATGGAGTACCCATACGACGTAC	Glysoja.06G016367.1
3301-35S-HA-GsMYST1-*Pml* IR	GGTCACCTGTAATTCACACTCAGTGGTGGTGGTGGTGGTGC	
GFP-*Bam* HIF	CTCCTTATAAAGAACAAAGTTCGGATCCCAGTAAAGGAGAAGAACTTTTCACTGGAG	
GsMYST1-GFP-R	CTCCAGTGAAAAGTTCTTCTCCTTTACTGGGATCCGAACTTTGTTCTTTATAAGGAG	
GsMYST1pro-GUS-*Eco* RIF	AGCTATGACCATGATTACGAATTCAATAAATAACCAATCAAAACATCACTTTATTTTCTAAAATATCAT	Glysoja.06G016367.1
GsMYST1pro-GUS-*Nco* IR	AGAAATTTACCCTCAGATCTACCATGGTAGAGAGACAGAGGGAGAGAAA	
pUC19-HA-GsMYST1-GFP-*Nde* IF	TGTTCCTGATTACGCTCATATGGGTTCACTCGAGGCC	
pUC19-HA-GsMYST1-GFP-*Sac* IR	CCTTGCTCACCATGAGCTCACTTTGTTCTTTATAAGGAGTCCAGATCAG	
pUC19-HA-GsNAC83-GFP-*Nde* IF	TGTTCCTGATTACGCTCATATGGAAAAGCTGAATTTTGTTAAAAATGGAGT	Glysoja.05G012860.1
pUC19-HA-GsNAC83-GFP-*Sac* IR	CCTTGCTCACCATGAGCTCAAAATGGGCATAGCCACTTGTT	
AD-GsNAC83-*Sma* IF	GGCCAGTGAATTCCACCCGGGGGAAAAGCTGAATTTTGTTAAAAATGGAG	
AD-GsNAC83-*Sma* IR	CCGTATCGATGCCCACCCGGGCTAAAAATGGGCATAGCCACTTGT	
GsMYST1-qPCR-F	CAAAGGAAAGGCTATGGCAAAT	Glysoja.06G016367.1
GsMYST1-qPCR-R	AGCAGTCCAAGGTCAGAAAG	
GsSnRK1-qPCR-F	TGCGTGATGGTCACTTTCTTA	Glysoja.13G035870.1
GsSnRK1-qPCR-R	CACAGCTCCAGACATCTACTTC	
GsNAC83-qPCR-F	GAACGACAAAGTGTGGCTATTG	Glysoja.05G012860.1
GsNAC83-qPCR-R	CAGTGAGTTCTGGAGCCTTT	
Bar-F	TGCCAGTTCCCGTGCTTGAA	
Bar-R	CTGCACCATCGTCAACCACTA	
GmACTIN-F	AACTGTGCGTGGCTACAA	Glyma.06G275200.1
GmACTIN-R	ATCCTTCACTCTCACCTTTCAC	
GsGADPH-qPCR-F	GACTGGTATGGCATTCCGTGT	Glysoja.06G014967.1
GsGADPH-qPCR-R	GCCCTCTGATTCCTCCTTGA	
GmCOR15B-A-qPCR-F	TGACCGGTGGAAGACATCTA	Glyma.17G040800.1
GmCOR15B-A-qPCR-R	TGCATTCAGTGAGGTATGCAA	
GmCOR15B-B-qPCR-F	CCACCACACTTGCATTGTATTT	
GmCOR15B-B-qPCR-R	CACCCTCGAAGCCATGATATT	
GmCOR15B-C-qPCR-F	ACCCTTAATGTTTGGTCCTATGT	
GmCOR15B-C-qPCR-R	GAGATAAAGGGTGTCATCCGAATA	
GmCOR15B-D-qPCR-F	TACAGAGAAGGCCAGAGAAATG	
GmCOR15B-D-qPCR-R	CCGGTGTTGTCCACCTAAT	
GmRD29A-qPCR-F	GAGCTGTTCAAAGTGAAGTAC	Glyma.10G236000
GmRD29A-qPCR-R	GTTATACACATGACCACGATCAA	
GmNADP-ME-qPCR-F	GGCCTTGAGAGATGGAGAG	Glyma.05G227400.3
GmNADP-ME-qPCR-R	CCCTCTCTTTCTCAGTGAAAG	
GmH^+-ATPase-F	GGACAAGGGAACCATCACTCTTG	Glyma.15G162600
GmH^+-ATPase-R	CAAGAAACTTGAGAAACTTGCTTTC	

Table S2

Motif Name	Sequence	Strand	Function
as-1	TGACG	-	unknown
ATC-motif	AGTAATCT	+	part of a conserved DNA module involved in light responsiveness
AT~ABRE	TACGTGTC	+	*cis*-acting element involved in the abscisic acid responsiveness
AuxRR-core	GGTCCAT	+	*cis*-acting regulatory element involved in auxin responsiveness
CCAAT-box	CAACGG	+	MYBHv1 binding site
CGTCA-motif	CGTCA	+	*cis*-acting regulatory element involved in the MeJA-responsiveness
GT1-motif	GGTTAA	+	light responsive element
LAMP-element	CTTTATCA	-	part of a light responsive element
TGACG-motif	TGACG	-	*cis*-acting regulatory element involved in the MeJA-responsiveness
WRE3	CCACCT	-	unknown

Table S3. Prediction of phosphorylation sites on GsMYST1 protein by GsSnRK1.

Name	Position	Peptide	Risk-Diff.
**GsMYST1**	43	KRRR**S**SVLP	3.21
	44	RRRS**S**VLPL	6.32
	292	KEKH**S**EESY	3.36
	338	ERPL**S**DLGL	3.17
	365	KGNI**S**IKEL	3.89

Table S4. Prediction of phosphorylation sites on GsNAC83 protein by GsSnRK1

Name	Position	Peptide	Risk-Diff.
**GsNAC83**	104	KRISSSTST	2.17
	105	RISSSTSTC	3.53
	143	YRLVSVETG	3.86
	173	MKKRSVESD	3.05
